# Shifting perceptions, preferences and practices in the African fruit trade: the case of African plum (*Dacryodes edulis*) in different cultural and urbanization contexts in Cameroon

**DOI:** 10.1186/s13002-021-00488-3

**Published:** 2021-11-08

**Authors:** Aurore Rimlinger, Jérôme Duminil, Taïna Lemoine, Marie-Louise Avana, Armel Chakocha, Alexis Gakwavu, Franca Mboujda, Mélanie Tsogo, Marlène Elias, Stéphanie M. Carrière

**Affiliations:** 1 SENS, IRD, CIRAD, Univ Paul Valery Montpellier 3, Univ Montpellier, Montpellier, France; 2 DIADE, Univ Montpellier, CIRAD, IRD, BP 64501, 34394 Montpellier, France; 3 Institute of Geography and Sustainability, Faculty of Geosciences and Environment, University of Lausanne, 1015 Lausanne, Switzerland; 4 CEFE Univ Montpellier - CNRS - EPHE - IRD - Univ Paul Valéry, 34293 Montpellier, France; 5 Faculty of Agronomy and Agricultural Sciences, Forestry Department, University of Dschang, BP 222, Dschang, Cameroon; 6 University of Yaoundé 1, Yaoundé, Cameroon; 7 The Alliance of Bioversity International and CIAT, Via di San Domenico, 1, 00153 Rome, Italy

**Keywords:** Smallholder agriculture, Sub-Saharan Africa, Tree crop, Agrobiodiversity conservation, Agroforestry, Urbanization

## Abstract

**Background:**

Understanding the perceptions, preferences and management practices associated with intraspecific variability of emblematic African tree crops is critical for their sustainable management. In this paper, we examine how the agrobiodiversity of a fruit tree species native to Central Africa, the African plum tree (*Dacryodes edulis*), is perceived and managed by Cameroonian cultivators.

**Methods:**

Semi-structured interviews and tree surveys were conducted over four months with 441 African plum tree owners from three different ethnic groups (Bamileke, Bassa, Beti) in urban, peri-urban and rural areas. Questions focused on trees owners’ perceptions—including the local nomenclature—preferences and management practices related to African plum trees and their intraspecific agrobiodiversity.

**Results:**

Across the three ethnic groups in the study area, more than 300 different local varietal names were recorded. These were mainly based on morphological and organoleptic traits, with two-thirds of the names referring to fruit size, skin color and fruit taste. The same traits were used by tree owners to describe their fruit preferences, but their relative importance in shaping fruit preferences varied among groups. The preferences of urban dwellers from different ethnic groups when purchasing African plum fruit focused on the fruit’s taste characteristics, while those of rural dwellers differed among ethnic groups. In rural areas, where African plums are sold and consumed by their growers, the preferences of Bassa consumers reflect quantity (fruit size) over quality (fruit taste or skin color) considerations. These preferences are reflected in the choice of seeds used for planting. Bassa owners sought seeds from trees with large fruits (with 34.8% of Bassa owners giving top priority to this trait as a selection criterion) to a significantly greater extent than Bamileke and Beti owners who prioritized taste and skin color instead. Among tree growers who selectively retained African plum trees in their fields, 44% considered tree productivity as a primary selection criterion.

**Conclusions:**

Findings linking perceptions of and preferences for fruit traits to intraspecific tree diversity, with attention to inter-ethnic and rural–urban differences, will help design locally specific measures to conserve the agrobiodiversity of African plum in the context of its ongoing domestication.

**Supplementary Information:**

The online version contains supplementary material available at 10.1186/s13002-021-00488-3.

## Background

Profound and rapid changes are currently taking place in global agricultural production and trade. In sub-Saharan Africa, changing eating habits associated with urban growth increase the demand for specific crops and agricultural products [1]. As such, rural cultivators need to adapt to potential changes in demand from urban dwellers [2], particularly for local crops and varieties with valued traits [3, 4]. Moreover, cultivators also consume a portion of the crops they grow, and their food preferences further guide their choice of cultivated crops and varieties [5]. The set of local crops and varieties they manage ultimately reflects demand, the varieties farmers value, as well as supply possibilities, the access they have to this set of varieties [6].

Understanding how farmers perceive their environment, and the nature and categorization of ecological perceptions as knowledge, meaning and preferences, is essential to understand the decisions agriculturalists make regarding species and agrobiodiversity management [7]. Within agrobiodiversity, which includes all the resources used for food and agriculture as well as the non-harvested species that support production [8], local varieties or ethnovarieties are defined as the intraspecific diversity, named and recognized by local users [9]. They are relevant entry points to understand how farmers perceive and manage their crop diversity [10, 11]. Perceptions—or cultural interpretations of sensory and biological information [12]—play a major role in farmers’ management decisions. Perceptions of crop traits vary across ethnic groups [13, 14]. Culturally specific names of local crop varieties reflect these different perceptions to some extent and provide insight into owners’ preferences, whether agronomic, aesthetic, or culinary [15, 16]. Preferences denote positive or negative individual evaluations of the objects under consideration, including comparisons or prioritization, and ranking [17]. Following the different perceptions they may have, diverse ethnic groups often favor other valued or unvalued traits [18, 19].

Emphasizing local perceptions and preferences of valued crop species also helps to show how they relate to practices. Research includes, for example, the link between local varietal knowledge and varietal management practices [20], between cultivator preferences and local variety conservation [21], and between cultivator values and on-farm variety persistence [22].

Changes in cultivators’ rationales and related patterns of crop diversity induced by market penetration and increased commercialization have received much attention [23–27]. There is also growing interest in traditional ecological knowledge of urban and peri-urban dwellers [28, 29], and numerous questions about the specificities of urban dwellers’ knowledge [30, 31]. Yet, there is a lack of scholarly attention to changes in knowledge—apprehended here through local perceptions and preferences—and practices, and their link with crop diversity along urbanization gradients, from rural agrosystems where crops are produced to urban areas where they are sold and consumed in large numbers.

In this study, we aim to document how perceptions, preferences and practices related to the agrobiodiversity of a cultivated African fruit tree species vary along an urbanization gradient and among three main ethnic groups involved in its trade in Cameroon. The fruit tree species *Dacryodes edulis* (G. Don) H.J. Lam (Burseraceae), locally known as the African plum tree (French: *safou*), is particularly important for the food security and economy of local populations in its native region in Central Africa [32–34]. This tree species is commonly found in agroforestry systems, home gardens and cities from Central Africa, where it is cultivated as a fruit and shade tree in coffee–cocoa agroforests and in home gardens [35]. It is a subdioecious species, some “male” trees presenting both male and hermaphrodite flowers in varying proportions, while “female” trees have only female flowers [36]. African plum trees are easily propagated from seed; vegetative propagation techniques have been developed by agricultural research centers to promote improved varieties [37] but are seldom known by cultivators [38]. Seed-propagated trees usually enter fructification five to eight years after planting, bearing fruits that are oval-shaped drupes, with high lipid, protein and fiber content [39]. Part of the staple diet and particularly appreciated, they can be eaten raw but are most often boiled or roasted to soften fruit pulp, and consumed with all types of side dishes [40, 41]. Fruit characteristics vary significantly between and within tree populations [38, 42–44]. Whereas African plum fruits have been tentatively classified on the basis of morphological traits [45] or morphological and biochemical traits [46, 47], cultivators’ perceptions of and preferences for the fruit have hardly been studied [48]. The ubiquity of the tree in urban, peri-urban and rural environments, where it is the most prevalent indigenous agroforestry tree species [49], makes it a good candidate for contrasting these perceptions and preferences across sites and ethnic groups. Moreover, consumer knowledge, perceptions and preferences for certain types of fruit are reflected in selling prices: in retail markets, large, thick-fleshed, good-tasting and oily fruits are sold at a higher price [33, 50]. Those fruits are thus more likely to be valued by producers.

Specifically, we examine how African plum trees owners view and use the species across an urbanization gradient, wherein urban areas are presumed to be predominantly consumption areas, and rural areas predominantly production areas. We then verify how these perceptions are corroborated by local names for the species, as local names often, but not only, refer to plant characteristics. Second, we observe how preferences for African plum vary according to fruit use (self-consumption, sale, purchase) and among differentiated groups of tree owners (rural and urban; from different ethnic groups). Finally, we examine patterns of variation in management practices, to see whether they reflect different preferences for the fruit. Following previous findings on end-users’ desired breeding traits in an African fruit tree species [19], we expected differentiated changes in the perceptions, preferences and practices in the context of rapid market changes along the urbanization gradient and between ethnic groups. We assumed more diversity in perceptions and practices in rural than in urban areas. As for the ethnic dimension, having no prior knowledge of ethnic-species links, we were keen to consider this dimension, which is structuring in many similar cases, even in the absence of specific prior assumptions.

## Methods

### Study site

We chose as our study area a region where cultivated African plum trees are abundant, both historically and due to a booming commercial production, as the region supplies Yaoundé (3.9 million inhabitants), one of the largest urban markets in Cameroon. Our sampling considered two parameters: (i) the continuous presence of African plum tree populations in agrosystems along an urbanization gradient stretching from urban to peri-urban to rural areas; (ii) the existence of three main ethnic groups (Beti, Bassa, Bamileke) in each of the main production basins supplying Yaoundé, where they are also settled.

**Fig. 1:**
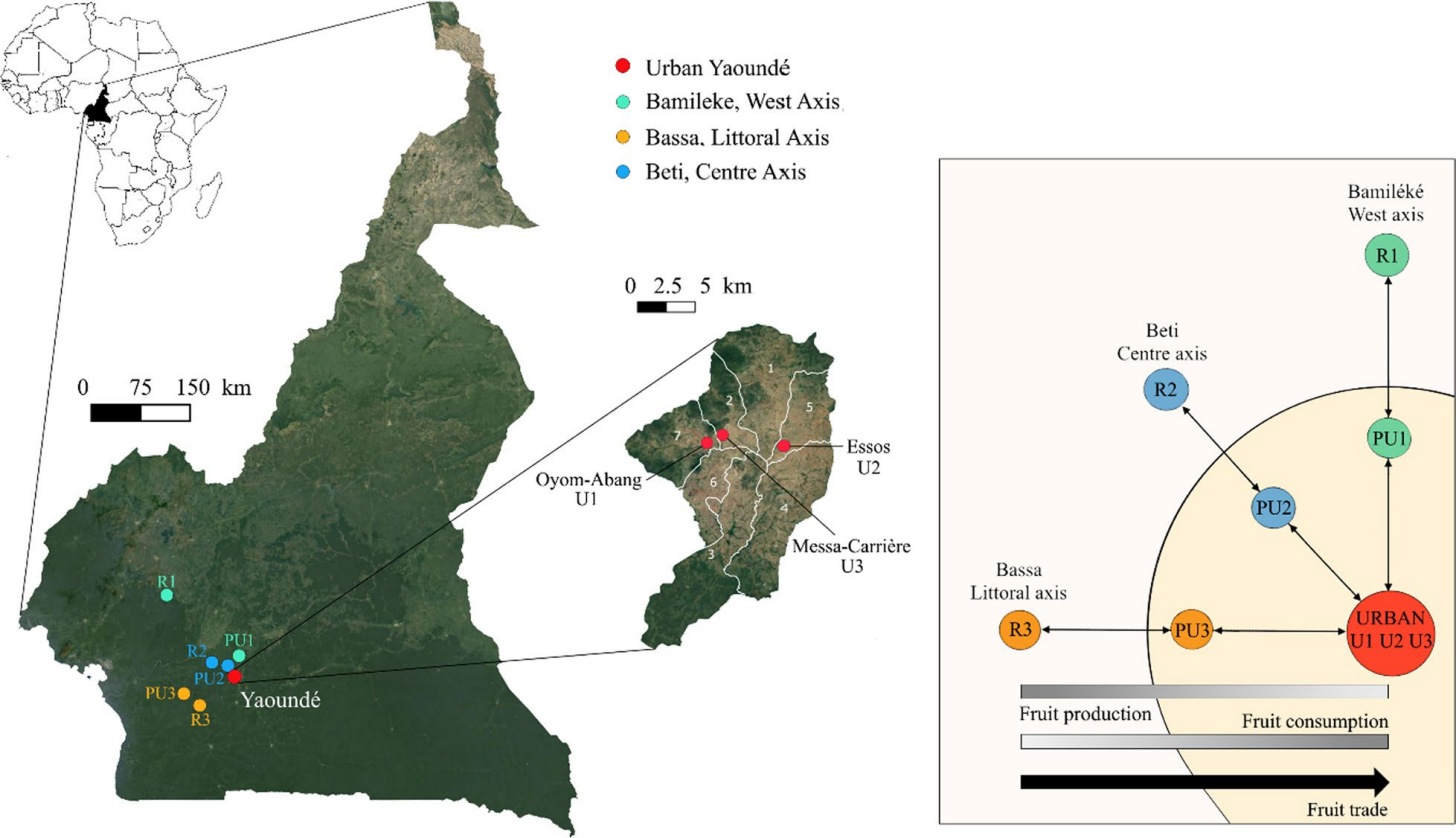
On the left, map of Cameroon showing the rural and peri-urban sites, with a close-up on Yaoundé for the urban sites. On the right, simplified representation of the urbanization gradient, going from urban sites to three rural sites passing through peri-urban sites, and of the distribution of the three ethnic groups (Bamileke, Bassa, Beti). Trends of fruit production and consumption are indicated by gray scales and fruit trade flows by a black arrow

Owing to the seasonality of production, the capital’s supply of African plum relies on different basins of production, populated by different ethnic groups [51]: the Littoral region (Bassa people), especially the Moungo and Sanaga departments which produce fruit at the beginning of the fruiting season (April, May); the Western region (Bamileke people) with the Noun and Ndé departments, as well as the neighboring region around Makénéné, where trees fruit in June and until the beginning of July; and the Central region located around Yaoundé (Beti people), with mature fruit produced in the Mbam, Lékié and Nyong Ekéllé departments from July to October. Study sites thus included: (a) urban sites, with three neighborhoods (Essos, Messa-Carrière and Oyom-Abang) in Yaoundé populated by both Beti, Bassa and Bamileke fruit tree owners with moderate housing density and buildings surrounded by small home gardens; (b) peri-urban sites, defined as agricultural spaces in which production systems are oriented toward urban market supply [52], with three towns close to Yaoundé and connected to it by asphalt road, one for each ethnic group (Beti : Okola ; Bassa : Eseka ; Bamileke : Obala, where many Bamileke people are settled); (c) rural sites, more detached from the urban market but still connected to it by trade, with three villages sampled, one for each ethnic group (Beti : Nkolekosting ; Bassa : Mbeng ; Bamileke : Bandounga ; Fig. 1). In rural sites, it is likely that some spontaneous African plum trees were occurring in low densities in surrounding forests, but as owners are not used to collect them in the wild, they were not considered in this study.

### Data collection

Semi-structured interviews were conducted with 441 plum tree owners from April to July 2018 (Table 1). Interviews were conducted in French and based on an opportunistic sampling with owners who would then point to possible respondents (snowballing approach). We collected information on the status of *D. edulis* tree owners (age and gender, not used in this study) to keep a good representation of the different age categories and gender. We included in the analysis their ethnicity and their location on the gradient (urban, peri-urban or rural dwellers).

**Table 1:** Summary of *D. edulis* tree owner characteristics along the urbanization gradient

Ethnicity	Gradient			
	Urban (*N* = 173)	Peri-urban (*N* = 127)	Rural (*N* = 141)	Total (*N* = 441)
Bamileke (Men/Women)	60 (24/36)	27 (16/11)	48 (34/14)	135 (74/61)
Bassa	37 (10/27)	50 (31/19)	44 (34/10)	131 (75/56)
Beti	76 (37/39)	50 (30/20)	49 (25/24)	175 (92/83)

Prior to the interview, tree owners were informed of the research intentions and of their right to participate or decline. At the end of the interview, tree owners were given a form stating that the interview had been conducted in accordance with the principles of free and informed consent, which they could sign if they agreed.

To document perceptions, the semi-structured interviews focused on two elements. First, we asked tree owners about criteria (including fruit traits) they used to distinguish African plum trees and their ethnovarieties. To see if similar traits were used in the local nomenclature of the three ethnic groups, we recorded the local names of African plum trees used by tree owners, their translation and their description. The described ethnovarieties were then categorized according either to the trait justifying their names, or to other justifications (symbolic, environmental, agronomic).

To describe fruit morphological characteristics, an identification sheet was used for tree surveys (Additional file 1), with several choices proposed for fruit size, shape, skin and pulp color. Fruit taste was also described orally, most often on a scale between very sour and very good, and later grouped in three standard categories: (very) good, average and (very) bad. To have the largest pool possible, we recorded both planted ethnovarieties (corresponding to the trees they had in their home garden or field, 806 trees in total) and known ethnovarieties (exercise based on the trees from other places the owners remembered, 427 trees in total).

To document preferences, the semi-structured interviews focused on the traits tree owners used to rank African plum trees and ethnovarieties. We asked with open-ended questions about their criteria for three different types of uses: self-consumption, sale (which types of fruits owners favored to be sold) and purchase (which types of fruits owners preferred to buy) of the fruit. We also asked about criteria that negatively affected the appreciation of the fruit (selection criteria). These different criteria were compiled for each owner and used as qualitative variables. Owners also reported their preference for each ethnovariety (“valued”, “not valued”).

To document practices, we collected information on how tree owners managed their trees. Some of these practices had a direct impact on the diversity of ethnovarieties (e.g., tree propagation and felling practices). Other practices (e.g., production techniques and traditional practices) were identified to determine whether they differed according to the location on the rural–urban gradient and ethnicity of tree owners.

### Data analysis

Differences between quantitative variables and tree owners’ groups (Table 2) were tested using a one-way ANOVA (for categories with more than two options) or a Wilcoxon ranking test. To see preferential associations between qualitative variables, cross-tabulation analyses were based on the χ^2^ statistic. The χ^2^ gives a global diagnosis of the dependence or independence between the variables. In order to visualize the categorical associations, mosaic plots [53] were plotted: The relative frequencies of the two variables are indicated by rectangles whose area is proportional to the cell value. The rectangles are colored if the residuals derive significantly from the independence hypothesis and are thus informative of the over- or under-representation of the association between some groups and variables. Analyses were carried out on R version 3.6.0 [54] using the vcd package, with the *shading_max* option [55].

**Table 2:** Data recorded during interview with tree owners and variables tested

**Qualitative variables**—χ^2^ **analysis**	
Buying preference criteria Self-consumption preference criteria	Selling preference criteria Planting preference criteria Selection criteria
**Quantitative variables—ANOVA**	
Number of used propagation techniques (e.g., seed directly sown, seed sown in nursery, graft-propagation) Number of traditional practices	Number of used production techniques (e.g., tree pruning, notching, use of fertilizers, use of pesticides)

To map how fruit traits were related to preferences, multiple correspondence analysis (MCA) was used. It was based on the data set combining the full description of ethnovarieties with fruit size, skin and pulp color and fruit taste. MCA is a method of factoring categorical variables (fruit characteristics) and displaying their associations in a two- or more-dimensional space. Owners’ preferences were not used as a variable in the analysis but were displayed within the same space as a supplementary variable: In this way, preferences associated with ethnovarieties were plotted on top of the ethnovarieties’ categorical dependent variables. The MCA was performed using the function “dudi.acm” present in the package “ade4” [56].

## Results

African plums were predominantly self-consumed, sold and purchased by tree owners. Food was the main use cited by more than 95% of the tree owners, the oily mesocarp of African plums being consumed after fruit boiling, grilling or roasting, by 100% of owners. Fruits that were consumed either came from privately owned trees or from fruits bought on the market, which 70% of tree owners reported doing sometimes. An even larger proportion of owners also sold them (78%). African plum tree owners gave an average price, for fruits sold in retail markets, of circa 1000 CFA (~ 1$80) per kilogram, although prices were said to fluctuate heavily depending on fructification seasonality and fruit size.

Other marginal uses of the trees were cited: medicine, with the use of leaves and bark for the treatment of many different diseases, and agronomy, as a shade tree in agroforests, accounting for 4% and 2% of the uses respectively. The average number of plum trees per owner was 1.4 ± 0.1, 13.1 ± 2.4 and 26.9 ± 2.8 in the urban, peri-urban and rural sites, respectively. Tree uses varied greatly along the gradient. In the urban sites, the dominant use was consumption or gift (84% of owners), while sale was marginal (16%). In the peri-urban sites, owners using exclusively their fruits for own-consumption accounted for 49% of the total, and 51% consumed some of the fruits and sold the rest to supply the urban market. In rural sites, most tree owners reported selling their fruits (83%). The proportions of exclusive consumers and sellers were similar in the urban and rural sites for the different ethnic groups, but in the peri-urban sites Beti owners were significantly more engaged in the plum trade (80% of them, *p* value < 0.001), compared to Bamileke and Bassa owners (33% of owners for both). Over the whole gradient, Bassa owned more trees (21.6 ± 3.1) than Bamileke (11.2 ± 2.1) and Beti (7.6 ± 1.1).

### Patterns of perceptions along the urbanization gradient and among ethnic groups

More than ten criteria for distinguishing African plum fruits and trees were recorded (837 citations in total, Table 3). Overall, the most cited criteria were fruit traits rather than tree features. The first criterion of distinction cited was the taste of the fruit (30% of the citation). The most cited taste was sourness, with good fruits being perceived as the least sour. The peculiar taste of African plum was expressed by the use of adjectives such as savory, tasty and fragrant (although these were mentioned by only five people). Taste descriptors also referred to texture characteristics: because of its soft/tender/smooth texture, it was compared to other foods, such as buttery avocado or dairy products (butter, cream, cheese). Its floury texture was mentioned as well; hence, it was compared with tubers (cassava, cocoyam, yam, baked potato). African plums trees were also distinguished by the way trees produce fruits, based on their productivity or their belonging to improved varieties.

**Table 3:** Distinguishing criteria of African plum trees cited by tree owners (*N* = 350)

Type of distinction criteria	Distinction criteria	Number of citations	Frequency of citations
Fruit traits	Taste	250	0.30
	Fruit size	231	0.28
	Skin color	162	0.19
	Fruit shape	80	0.10
	Pulp color	60	0.07
	Pulp texture	22	0.026
	Pulp width	7	0.008
	Skin quality	4	0.005
Specific tree features	Tree productivity	15	0.018
	Leaf size	2	0.002
	Others	4	0.005
Total		837	1

**Fig. 2:**
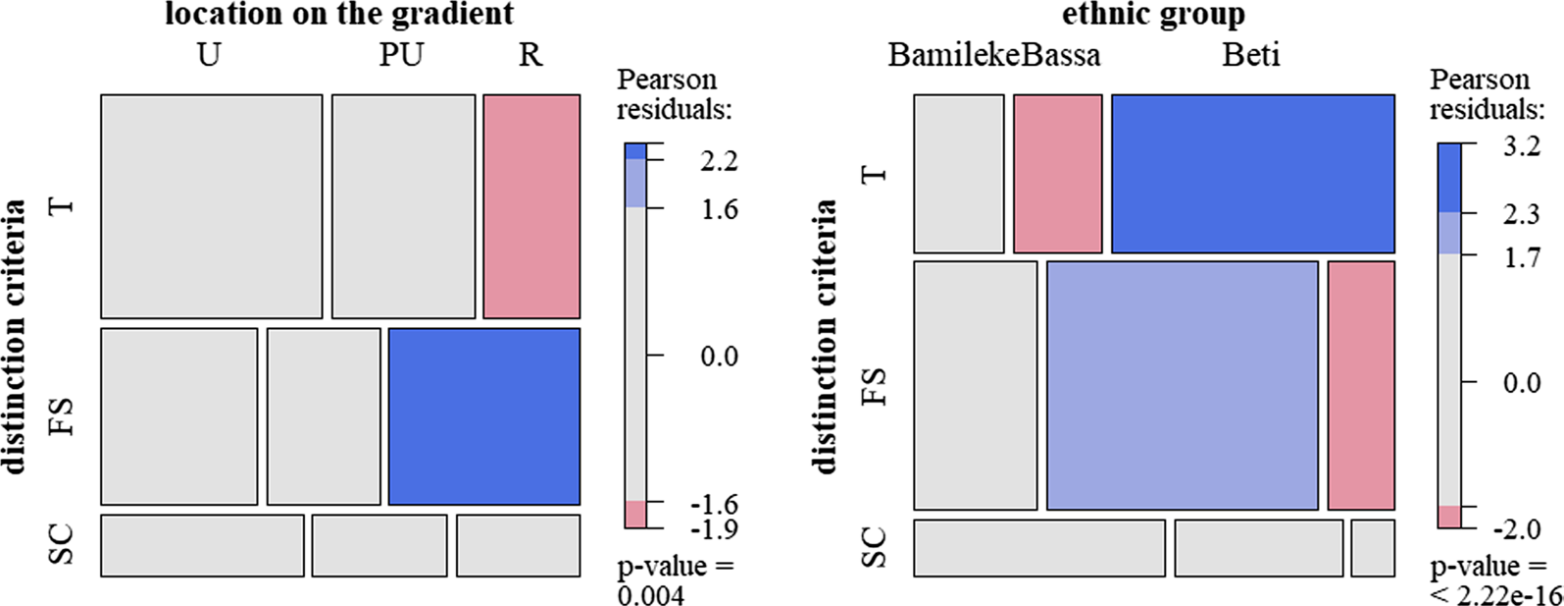
Mosaic plot showing associations between tree owners from different sites (U = urban, PU = peri-urban, R = rural, *N *= 307) and between different ethnic groups (Bamileke, Bassa, Beti) in rural sites only (*N* = 91), and the three most cited criteria; T: taste, FS: fruit size, SC: skin color. The color corresponds to Pearson residuals from the χ^2^ test: red for the under-represented intersections (*p* < 0.05), blue for the over-represented ones and gray for those that are close to the values expected in the independence hypothesis

The three most common criteria (taste, fruit size and skin color), which accounted for more than 75% of the total responses, were not cited with similar proportions along the gradient. To distinguish plum varieties, rural dwellers cited fruit taste less often, but instead cited fruit size as their first criterion (47.6% of citations by rural dwellers, *p* value = 0.008; Fig. 2). Differences in the frequencies of traits cited by the three ethnic groups to distinguish plums were mainly found for the rural site. The trait most cited by rural dwellers, fruit size, was significantly more cited by the Bassa owners, who accounted for 59% of fruit size citations.

**Fig. 3:**
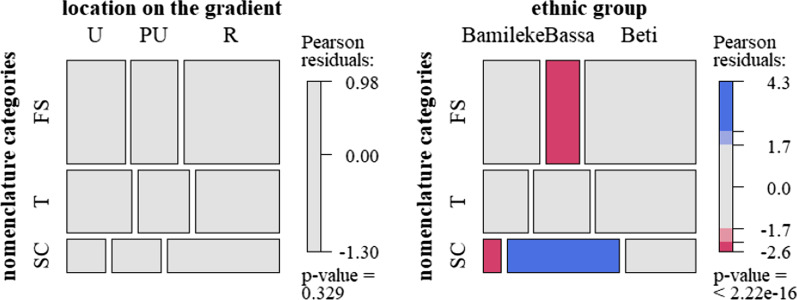
Mosaic plot showing associations between tree owners from different sites and between different ethnic groups (Bamileke, Bassa, Beti, *N *= 341) in all the sites combined, and the three criteria mainly used to name ethnovarieties, FS: fruit size, T: taste, SC: skin color

Of all the 1083 trees described, almost half (526 trees) had been given a local name by its users, whereas no name was recorded for the rest of the trees (51%). Most names were recorded in rural sites (269 trees), followed by peri-urban and urban sites (126 and 131 names, respectively). As regards the distribution by ethnic groups, 303 (58%) of these trees were named by Beti, 124 (24%) by Bassa and 99 (19%) by Bamileke owners in their respective languages. Due to the absence of a common Bamileke dialect, Bamileke names were rarely shared, except at the rural level. Within one ethnic group, different names were also recorded for fruits with similar traits. Names were not shared between ethnic groups, even in urban sites where ethnic groups cohabit closely. However, the meanings of these names were shared across groups (Fig. 3). Ethnovarieties named using these three different traits were cited with similar proportions along the gradient. Among ethnic groups, Bassa owners named significantly fewer varieties based on fruit size, and more based on skin color.

**Fig. 4:**
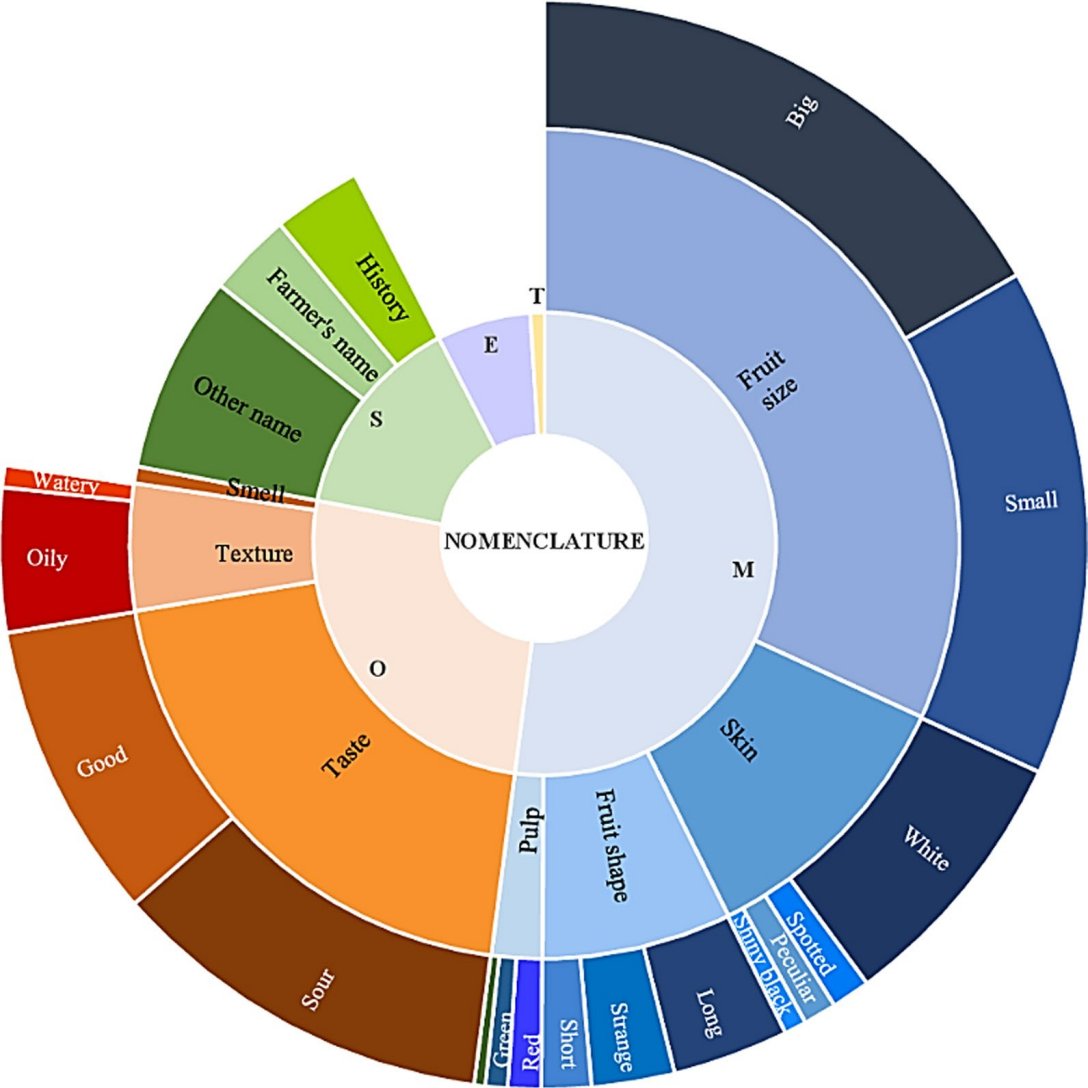
Sunburst graph showing the nomenclature of African plums with the different categories of criteria in the graph center and the subcategories in the graph edges, following the number of citations of each (sub)categories. M: morphological, O: organoleptic, S: symbolic, E: environment, T: type of tree (“natural” vs “improved” tree)

As some names were cited several times (180 were cited by at least two respondents), a total of 346 different African plum tree local names were recorded from 219 different tree owners. Regardless of ethnic group, names mainly referred to morphological (size, skin color, shape) and organoleptic (taste, texture) criteria of the fruit (78% of the names), followed by names referring to the history of the tree, including its owner’s name or the place where the tree was planted (Fig. 4). Ethnovarieties were most often named based on their fruit size (37% of the names), taste (20%), and skin color (11%), which were also the three most cited traits for distinguishing fruits. Some ethnovarieties were recognized and named based on the combination of several of these criteria, fruit size and taste or fruit size and pulp color, for instance.

### Patterns of uses and preferences along the urbanization gradient and among ethnic groups

African plum trees owners reported the use of different parts of their trees. The use of fruit as food (self-consumption) was reported by all plum tree owners throughout the gradient, whereas its use as a commodity varied along the gradient: Fruit sales increased from urban to rural sites. The second most used part was tree branches (55% of owners), which were collected as fuel wood for cooking. The use of its bark (34% of owners) and leaves (33% of owners) were reported, both for medicinal purposes. The tree as a whole was also reported to be useful by 10% of the owners, for the shade it provided to cocoa and coffee trees in agroforests. Finally, few owners (2%) cited roots as a part of the tree they used, also for medicinal purposes. These uses were unrelated to preferences for most tree owners, but some reported preferences for medicinal uses. These preferences concerned trees bearing rare white fruits (“white plum trees are more used for indigenous remedies; it is also the case with white cola and red corn” Obala, Eton owner, May 2018), the bark and leaves of male trees that never bear fruits (*nèlom sa,* Bassa) to prepare remedies (bark for tuberculosis, leaves for jaundice) or specific age categories of trees (bark and leaves of old trees used to prevent typhoid fever; bark and leaves of young trees used to prepare treatment for snakebites).

We recorded the preferences that were associated with the main uses of the fruit. Overall, eight criteria were primarily (99%) cited to describe preferences, whether they were related to self-consumption, purchase or sale. Seven out of these nine criteria were related to fruit traits: taste, fruit size, skin color, pulp texture, pulp color, pulp width and fruit shape. For some people, the geographic origin of African plums was also one of the most important criteria of choice. Other criteria (smell, seed color, affinity with a seller) accounted for less than one percent of the total citations. Other owners (9%) reported to have no criteria, meaning they liked all fruit types equally.

**Fig. 5:**
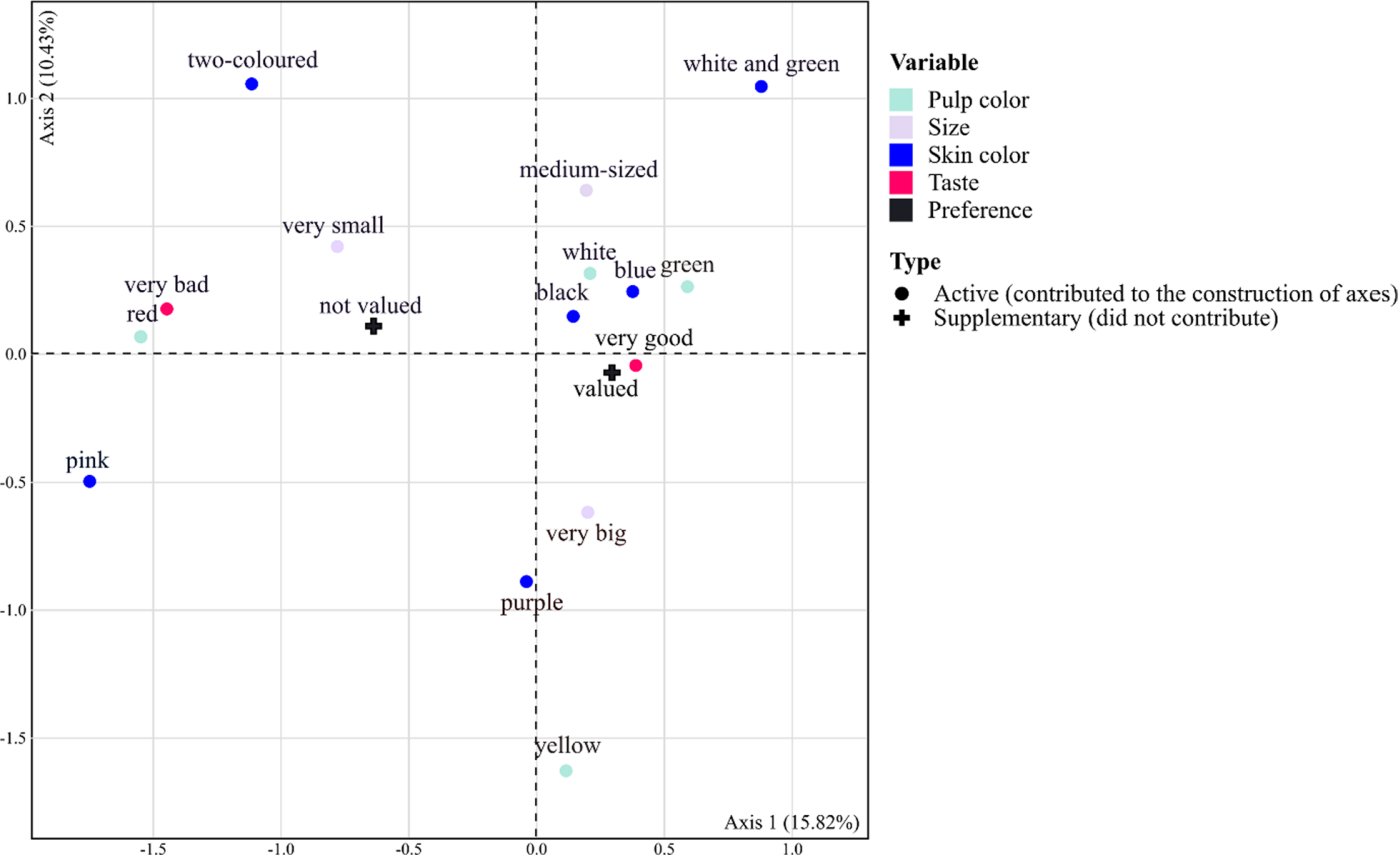
MCA discriminating different groups of ethnovarieties based on pulp and skin color, fruit size and taste, with as a supplementary (inactive) variable the preference criteria relative to the ethnovarieties (valued or not valued). Fruit traits on the right side of the graph correspond to the valued ethnovarieties, fruit traits on the left side of the graph correspond to the non-valued ethnovarieties

Globally, tree owners described the ethnovarieties they knew or had planted with four main criteria (pulp and skin color, size and taste of the fruit) and they specified if they liked or disliked them (Fig. 5). The valued ethnovarieties were those with a blue, black, white or green skin, a green or white pulp, a medium or big size and a mildly sour taste (traits close to the “valued” preference type). The ethnovarieties that were not valued were those with a pink or a two-colored skin, a red pulp, a very small size and a very sour taste (traits close to the “not valued” preference type).

Irrespective of whether the fruit was used for self-consumption, purchase or sale, the most valued traits for selecting fruits were its taste, size and skin color. The three criteria taken altogether accounted for 94% of preference criteria. Some owners (12%) stated that they had no preference criteria and were eating/buying/selling all types of African plums equally.

**Fig. 6:**
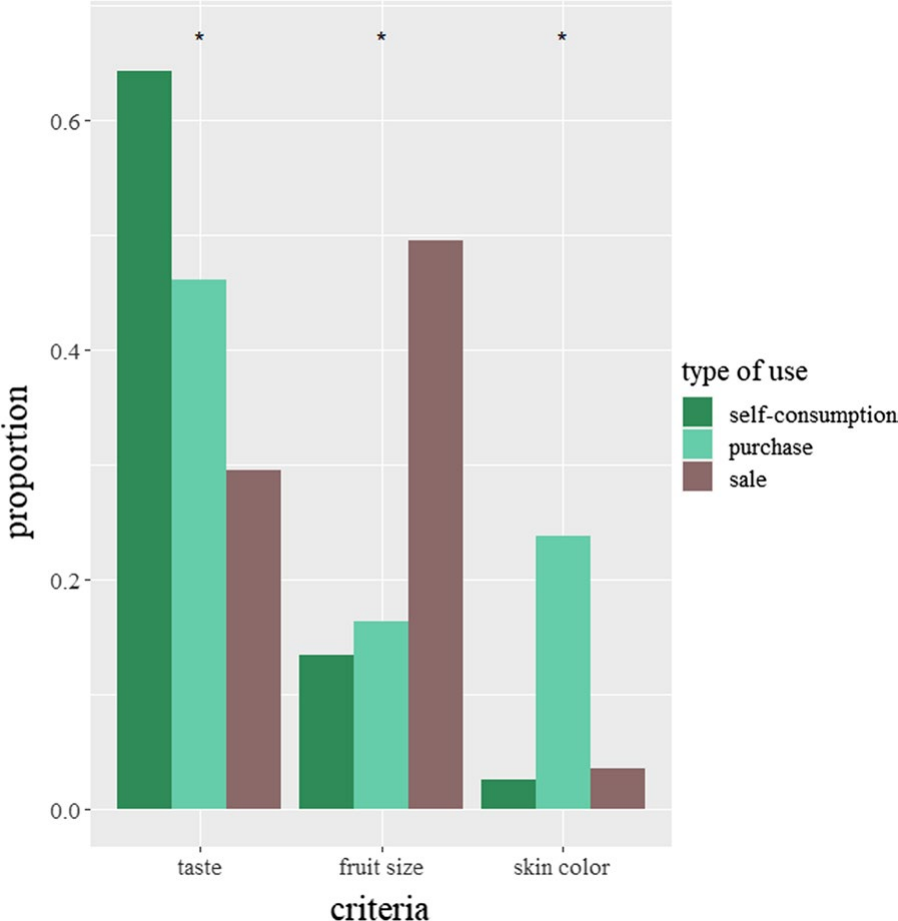
Bar plot showing the relative frequencies of the four most cited fruit criteria depending on the type of use considered. The stars signal criteria that were significantly different between categories of preferences

The favored criteria for fruit selection varied according to the types of uses (self-consumption, purchase, sale) considered (Fig. 6). Taste was the most cited criterion related to self-consumption and purchase of the fruit (64% and 46% of all cited criteria for these use types, respectively). For purchase preferences, taste was followed by the skin color criterion (24%), which was used as a proxy for taste: The darker the African plum, the better the taste. Fruit size was the most cited criterion (50%) of the selling preferences, with large fruits getting to be sold and small fruits kept for self-consumption.

**Fig. 7:**
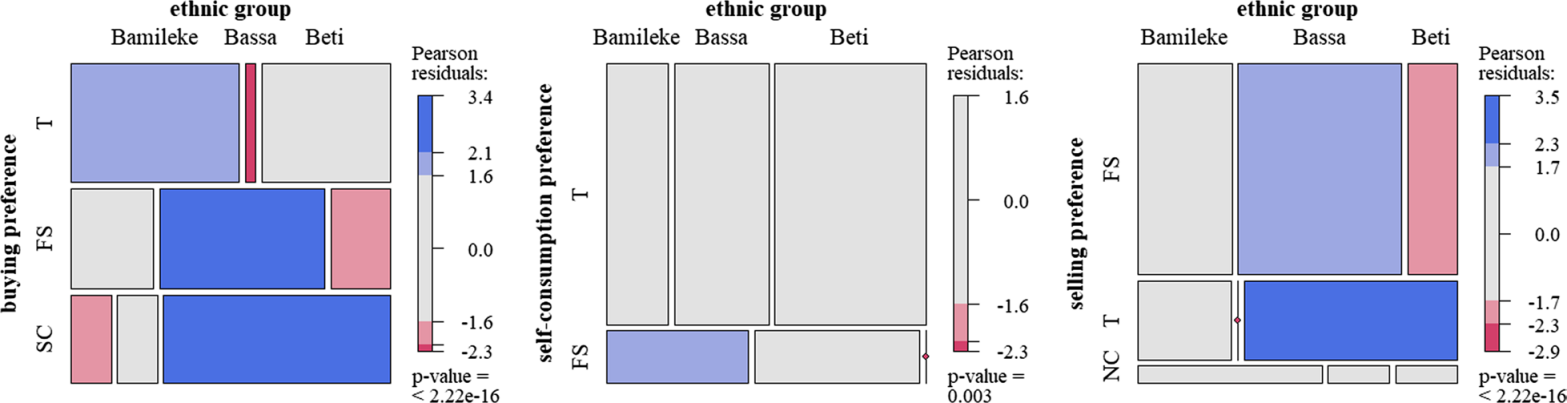
Mosaic plot showing fruit traits (T: taste, SC: skin color, S: fruit size, NC: no criterion) cited by owners from different ethnic groups in rural sites for different types of uses, from left to right: purchase (*N *= 80), self-consumption (*N *= 100) and sale (*N *= 95)

As for preferences, the most frequently cited criteria were always the same across ethnic groups for urban sites, but differed significantly across ethnic groups for rural sites (Fig. 7). Fruit size was over-represented as the primary preference criterion for the following groups and preference categories: Bamileke owners for self-consumption (35% of Bamileke owners, *p* value < 0.001); Bassa owners for buying and selling (respectively, 64% and 87% of Bassa owners, both *p* value < 0.001). Beti owners cited fruit taste more often for selling preferences (50% of Beti owners), emphasizing the importance of selling non-sour fruits.

### Management practices along the urbanization gradient and among ethnic groups

Two categories of management practices (propagation practices, cultivation practices) were recorded. Some of them were aimed at obtaining valued types or at getting rid of undesirable trees (propagation, counter-selection), some were related to other aspects of management (production techniques such as pruning of trees, use of fertilizers).

Propagation practices included tree transplanting, direct seeding in the field, or in a backyard nursery and later transplanting the plant into the field. Vegetative propagation was generally not used. Production techniques either targeted individual African plum trees (pruning, heading) or the whole field (natural and chemical manure application, watering, weeding). Overall, more propagation and cultivation practices were cited (one-way ANOVA, p < 2.98e^−05^, Table 4) in rural (4.4) and peri-urban sites (4.2) than in urban ones (3.6). Bamileke owners cited more propagation and cultivation practices (4.9) than owners from other ethnic groups.

**Table 4:** African plum tree propagation and cultivation practices reported by tree owners of different ethnic groups along the gradient (means ± sem)

	Location on the gradient								
	Urban (*N* =173)			Peri-urban (*N* = 127)			Rural (*N* = 141)		
Number of propagation practices	1.7 ± 0.07^a^			2.0 ± 0.08^b^			2.2 ± 0.07^b^		
	Bamileke	Bassa	Beti	Bamileke	Bassa	Beti	Bamileke	Bassa	Beti
	1.9 ± 0.1	1.6 ± 0.1	1.6 ± 0.1	2.0 ± 0.2	2.1 ± 0.2	2.0 ± 0.1	2.1 ± 0.1^b^	2.6 ± 0.2^a^	2.0 ± 0.1^b^
Number of production techniques	1.9 ± 0.1			2.2 ± 0.1			2.2 ± 0.1		
	Bamileke	Bassa	Beti	Bamileke	Bassa	Beti	Bamileke	Bassa	Beti
	2.3 ± 0.2^a^	1.7 ± 0.2^ab^	1.6 ± 0.2^b^	3.0 ± 0.2^a^	2.0 ± 0.2^b^	1.9 ± 0.2^b^	3.6 ± 0.2^a^	1.6 ± 0.1^b^	1.3 ± 0.1^b^

Means in a row without a common superscript letter (a, b or both) differ (*p* < 0.05) as analyzed by one-way ANOVA and the Tukey’s test

Practices that were not linked to the management of tree varietal diversity were also recorded. Traditional practices were used by 14.1% of the owners to manage low production or high fruit drop. The two most common practices were notching the trunk with a sharp object (6.9%) and tying the trunk with lianas or banana leaves (6.3%). Bassa tree owners (19.7%), followed by Bamileke owners (15.3%) cited these practices, whereas Beti owners (10.0%) applied them much less frequently (*p* value = 0.038). Urban dwellers also cited far fewer practices (8.7%, *p* value = 0.035) than did owners living in peri-urban (17.1%) and rural (17.5%) sites.

**Fig. 8:**
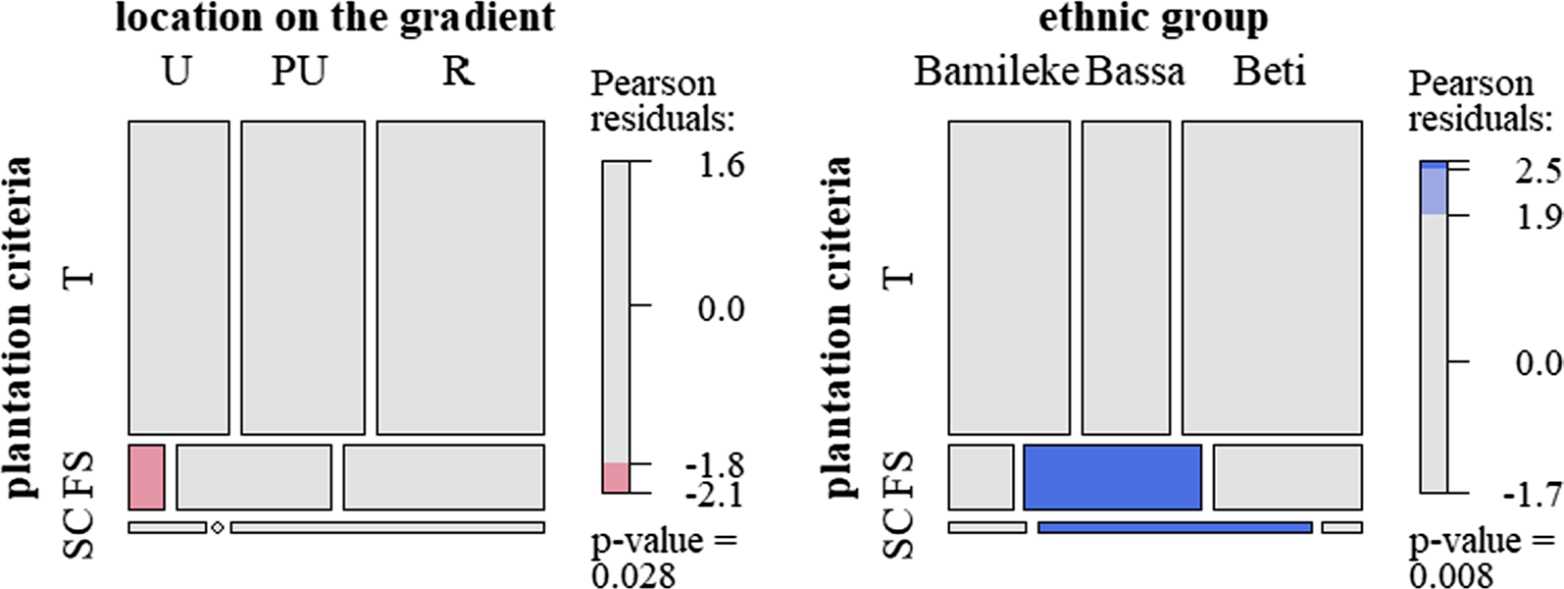
Mosaic plot showing planting choices (T: taste, FS: fruit size, SC: skin or pulp color), according to gradient location and ethnic group in the rural sites (*N *= 344)

As for the seed chosen to be planted, two criteria (fruit taste and fruit size) represented more than 90% of the criteria cited. Some other owners (40%) used no criteria. Urban dwellers took much less account of fruit size (*p* value = 0.01) than did peri-urban and rural dwellers (Fig. 8). In the rural sites, Bassa owners cited fruit size (34.8% of the Bassa's first planting selection criteria; *p* value < 0.001) significantly more than Bamileke and Beti owners (7% and 18.3%). They also cited skin color more than the other owners.

Most of the seeds selected for planting did not come from the owners' own fields (77% of the 467 trees with information on the seed provenance). They came mainly from trees in the village or city through exchanges with neighbors (14% of seed sources) or from fruits in the market (local or city market, 37%). Only a few trees from spontaneous regeneration were protected or transplanted (6%). The average distance between the seed source and the planting site was high in urban sites (55.1 ± 5.2 km), intermediate in peri-urban sites (18.9 ± 4.7 km) and low in rural sites (6.7 ± 1.3 km). In urban sites, seeds were brought back from the villages of origins of the tree owners and from regions known for the quality of their fruits (Makénéné market, improved varieties from the IRAD). This distance is underestimated because some seeds were bought at the market and therefore of unknown origin. The average distance is thus much greater in peri-urban and urban sites, where about 40% of the trees originated from market seeds.

Of the 23% of seeds that were coming from the owners’ field, owners were asked how they selected which tree to propagate. These trees were mostly (91%) owned by owners in peri-urban and rural sites; urban dwellers stated that they had too little space. The two main selection criteria for these seeds were the taste of the parent tree’s fruits, such as a mildly or very mildly sour taste (61%) and fruit size (29%), with large fruits being multiplied regardless of their taste. Only five percent of owners cited air-layering as a propagation practice that they knew or used: They were from all ethnic groups and evenly distributed along the gradient.

Undesirable traits further guided selection practices. Not all tree owners selected their trees; only 230 owners (52% of the total) did. Urban dwellers were significantly less likely to report selection practices (44% of them). Bamileke owners reported selection practices much more often (68% of Bamileke owners, *p* value < 0.001), and Beti owners significantly less (41% of them). A variety of criteria (six major categories of criteria) justified tree selection, some targeting specific undesirable traits. A problem cited by 20% of owners was that the desired traits of the mother tree were not inherited in its offspring, which made selection ineffective for achieving desired traits.

Lack of production (cited by 44% of the selective tree owners) leads to the felling of unproductive trees. This practice was cited more (*p* value < 0.001) by Bassa owners (63% of Bassa owners) than by Bamileke or Beti owners (38% and 36% respectively). Urbanization (15%) and the resulting lack of space for maintaining existing trees led to tree felling. This criterion was significantly (*p* value < 0.001) more cited in urban than in rural sites (representing 27% and 4% of the citations per site, respectively). Bad (sour) fruit taste led to the felling of some trees. This criterion was cited by 15.4% of selective owners with similar frequencies among tree owners throughout the gradient and across ethnic groups (18.3, 9.6 and 16.7% for Bamileke, Bassa and Beti, respectively). In the peri-urban and rural sites, excessive shade for cocoa trees sometimes resulted in cutting down plum trees (10.6% of respondents). Tall trees (8.1%) were felled because they were difficult and dangerous to harvest. Finally, some trees were felled (7.0%) because of their small fruit size.

## Discussion

### Ethnovariety-naming dynamics and evolving perceptions along the urban–rural gradient

A vast number of different names were recorded, in all three ethnic groups surveyed. This high number is in part due to synonymy, which occurs when fruits with similar traits are named differently. The lack of name consistency in varieties can stem from the presence of different languages [57], from significant variety exchanges [15] and from the acquisition, at the owner level, of “new” varieties (already present in other farms or villages) obtained through cross-pollination and named differently. Given the scarcity of the scientific literature referring to varietal names of fruit trees within the studied area, it is difficult to say how remarkable this result is. Nonetheless, comparison is possible with other fruit tree species cultivated in Central Africa. Imported fruit trees, which entered production systems later than indigenous species such as the African plum tree, could also have different names, but in limited number and without symbolic value. This is the case, for example, for mangoes and avocadoes, whose names qualify instead their origin (Ngaoundéré’s mango, German mango), their importance in local trade (“Number One” mango; [58]) or their texture (“butter avocado”). For the Beti people in particular, the nomenclature described for the perennial species *Musa* × *paradisiaca* was more expanded than for the previously investigated fruit trees [59], but was not as dense as what reported here for the African plum tree.

This extensive ethnovarietal nomenclature, which is also found for other African perennial species in East and West Africa [60–62], expresses the salience of the species [63], and the human involvement around it [64]. The great morphological differences between African plums foster the diversity of local names as well, with phenotypic boundaries being delineated by cultural preferences [65]. The names of ethnovarieties shared along the urbanization gradient for Beti owners also indicate the flow of information within communities through learning the general distinctive features of ethnovarieties and their associated names [66]. This, along with the fact that more than half of the names recorded originated from Beti owners, is quite a puzzle and begs for more investigation on the singular nature of this finding.

Linguistic expressions are indicators of the cultural and social dimensions and value of agricultural resource management: Through local names, significant elements of the cultural and economic importance of varieties, and of their commonness, are revealed [67]. It is thus worth noting the predominance of names referring to size in the local species nomenclature system. With regard to the variation in perceptions of the species (i.e., the way sensory and biological information regarding African plums are interpreted by their owners) along the gradient, the most common fruit traits used to define African plums differed. More rural dwellers cited fruit size as the primary criterion for distinguishing plums, whereas fruit taste was predominantly mentioned by urban dwellers. The dominant use of the fruits in the different sites, respectively, sales and self-consumption, was thus reflected in these two contrasted perceptions.

Perceptions also differed among ethnic groups in all sites except for urban sites where they were homogeneous. Rozzi [68] proposed that rural–urban migration, by bringing in cities a diversity of ethnic groups, favors knowledge homogenization. Even if plant knowledge loss is not necessarily occurring in urban areas [69], the homogenization of knowledge among different cultural groups could be a likely consequence of urbanization and has already been described regarding plant knowledge in urban markets [70]. In our case, the effect of having together different ethnic groups in urban environments might be acting on its nomenclature as well. Indeed, the names of ethnovarieties differ from one language to another (Bamileke, Bassa, Beti), and even within Bamileke, a linguistically fragmented language [71], leading to the sharing of local names only at the rural scale for this group. But with the marketing of African plums linking people from different ethnic groups and languages, it would be interesting to see to what extent ethnovarieties that have been given local names are renamed by people specialized in the African plum trade. The nomenclature of other food products was simplified in urban markets [72], with traditional names being adapted to optimize trade [73].

Overall, African plum tree is among the most important indigenous food tree in Central Africa, thanks to its use as both a food and a commodity. The tree has also a cultural value, expressed through its symbolic use as a living memory. Among the Beti, it was commonly said that plum trees were planted or dedicated to the memory and commemoration of exceptional people. This could be manifested by planting a seed from a parent (especially older or notable people), or by letting a young child plant the seed, leading to an association between the two. This association also exists when a tree is planted for a newborn [74]. It was seen as well in the fact that some trees are named after people, either the cultivator himself or other relatives. In southern Africa as well, fruit trees are often known by individual names, referring to community members [75]. An additional peculiarity distinguishes it from fruit trees: Theft of African plums, as well as that of kola nuts, would be punished by customary code [76, 77]. However, the cultural value of African plum was not manifested through ceremonial gift exchanges, unlike products from other indigenous fruits trees, such as *Garcinia kola* seeds [78], *Raphia* wine and products [79] and many others [80].

### A typology of preferences according to sites: from subsistence to market values

Ethnic groups had more similar preferences in urban sites than in rural sites. Regardless of the type of use considered (self-consumption, buying or selling), the fruit preference criteria were not significantly different between ethnic groups living in an urban environment. The urban specificity that might come into play is that owners are exclusively consumers, of their own fruits and of fruits bought on the market, rather than sellers.

The different rationales of consumers and sellers, studied through preferences related to the different uses of plums, showed that preferences changed in the rural, peri-urban and urban sites as a result of the different uses. Fruit taste was valued preferentially through self-consumption preferences and buying preferences, which are those of consumers. On the contrary, fruit size was ranked first in selling preferences. The shift from preferences based on a quality-related trait (fruit taste) expressed by urban dwellers, who are mostly consumers, to preferences based on a quantity-related trait (fruit size) expressed by rural dwellers, who are mostly sellers, shows the strong influence of market-driven logics. Although the preferences expressed by consumers (“buying preferences”) target fruit taste, the owners prefer to sell large fruits. These fruits are indeed more expensive on the market [81] and thus more profitable to the sellers. They are also more sought after by *buyam-sellam*, intermediaries who buy crops or non-timber forest products in bulk and then retail in urban markets [82] and who are in charge of a large part of the African plum trade. As they travel to rural environments to buy plums and transfer them to urban markets, their overall standards are less focused on quality [83].

In rural sites, where most owners are also sellers, the important value of plum size is corroborated by the fact that this trait is also overrepresented for preferences which are categorized as consumer preferences (self-consumption, buying), preferences that are never associated with size in urban sites. But the two uses (as self-consumed food or as a traded commodity) are not necessarily contradictory. Rural Beti owners, for instance, seem more attached to the criterion of taste than the other ethnic groups and even cite it when expressing their preferences as sellers. Incidentally, this logic is more in line with buying preferences, which are mainly based on fruit taste.

### Perceptions and preferences turned into practices

Analyzing the variation in perceptions and preferences is crucial as both reflect cultural or economic values and influence management practices. Owners indeed aim at increasing the phenotypes producing the desired fruits in the managed tree populations [84]. Here, following the valued traits and preferences, practices of rural and urban dwellers also differ. *Dacryodes edulis* is mainly propagated using seeds collected by farmers on trees presenting fruits with desirable traits. Urban dwellers are using the fruit size criterion less often than peri-urban and rural tree owners to select the tree to be propagated. The fruit size criterion is more cited by rural Bassa owners than Beti or Bamileke as a key trait for selecting seeds used to plant their trees. As Bassa owners also cited fruit size as a preferential buying criterion, this shows how preferences may be translated to some extent into planting practices. However, improved planting materials, which would be the best option for obtaining trees with the most valued characteristics, were used by only a few owners. The improved varieties (named cultivars) developed in agronomic centers [50] were indeed perceived by these owners as having valuable traits: small trees are easy to harvest, and large fruits easy to sell. But the improved cultivars were also described as having non-valued traits, being characterized as fragile and short-lived trees, with fruits whose taste tends to become more and more sour over the years.

Concerning selection practices, Vasquez and Gentry [85] described some attributes motivating, in the context of commercial trade, the felling of less profitable trees. They were related to: (i) the fruiting phenology of the species (dioecious species with separate male and female trees), (ii) fruit accessibility and (iii) demand for fruit. As for *D. edulis*, trees bear either female flowers only or a variable ratio of male and hermaphrodite flowers [36]. For this latter category, trees presenting a high proportion of male flowers tend to be removed. The African plum trees most likely to be felled are therefore (i) unproductive trees bearing mostly male flowers, (ii) large trees that are difficult to harvest and (iii) trees that bear the fruits least valued on the market (sour fruits, small fruits). But the low percentage (< 10%) of owners citing fruit size as a reason for selection also shows that market logic is not the only reason for choosing which trees to keep. Plum trees that bear small fruits are, for instance, often referred to as “the children’s plum tree”. In contrast to trees bearing large fruits, often preserved for the market or the family, these trees are used by children to collect the fruits without causing problems. The diversity of plums’ morphology and taste thus ensures that the uses and expectations of the species’ cultivators complement each other. Its maintenance acts as a strategy to maintain food security or reduce risks [38], as was seen for other tree species in sub-Saharan African [86]. More broadly, the diversity of local varieties can be valued in itself, as an object of pride for cultivators [87, 88].

### Implications for African plum agrobiodiversity

From our study, two sets of values are coexisting. On the one hand, owners who predominantly sell value a quantity-related trait and thus express that the increased market accessibility for large-sized plums is a strong incentive for them to grow trees of these varieties. Other studies have highlighted the predominant role played by economic and social drivers in the choices made by individual farmers between local varieties [89]. Farmers’ management is altered by changing market incentives due to the growing importance of certain products or varieties [90]. Farmers' tendency to produce specific crops and varieties that meet market demand is identified as one of many changes underway in sub-Saharan agricultural systems [91]. On the other hand, owners who predominantly consume value a quality-related trait, taste, which is found in all kinds of different ethnovarieties, and thus express a wide range of preferences. This finding is important to better understand that the ongoing domestication process does not always target the largest fruits, which are not necessarily the most sought after by consumers. This was also expressed when some owners answered that they had no criteria for the African plums they were planting: All types of ethnovarieties had the same value to them. Indeed, attributes valued by local markets often fail to explain the variation in the number of varieties grown on farms [92].

Our findings underscore the need to analyze several levels of knowledge (different ethnic groups, urban and rural dwellers) to obtain a comprehensive picture of the considerations underlying the diversification or erosion of varietal diversity. Urban centers can be important repositories of diversity, as urban owners are less concerned about selecting trees based on fruit size. Urban home gardens thus have multiple potentials as production and conservation areas, all the more as they represent unexpected places to safeguard species’ genetic diversity [93]. On the other hand, although more specific criteria are applied to planting material in rural environment than in urban and peri-urban ones, their effect is limited by the predominant allogamy of the species: Cross-pollination between trees, combined with high heterozygosity (high genetic variability), prevents the reproduction of targeted maternal traits. As selection practices are rather weak on trees that bear non-valued fruits, pressures on plum agrodiversity remains relatively low. The selective retention of these less useful trees in the future might also depend on land pressure, which is increasingly strong along the urbanization gradient.

The value of local varieties is assessed first and foremost by what farmers say and do about them, and is therefore based on their uses and expectations [8, 24, 94]. These uses respond to different needs and constraints, both for cultivators themselves (ecological benefits, consumption qualities, cultural significance) and for use on the market, in order to take advantage of commercial opportunities [95]. Uses and preferences are functionally related in that different traits valued for local varieties correspond to different uses [13, 96]. In our case, the main uses—self-consumption, sales—are, respectively, associated with fruit taste and fruit size. These preferences are translated into practices as rural owners who are also sellers are more likely to plant trees using seeds from large fruits. Market integration is expected to change the uses of local varieties, with a shift from subsistence to trade-oriented strategies, and to homogenize the valued traits of varieties [97]. For African plum trees in Cameroon, this risk is mitigated by the current exclusion of fruit crops from government food security policies, which therefore receive neither preferential treatment under subsidized seed dissemination programs nor extensive tree breeding programs. Competition between improved cultivars and traditional seed systems is thus weak. Besides, market development could go hand in hand with the development of new opportunities for local varieties [98].

## Conclusion

African plums are food products as well as commodities. Urban dwellers use them as the former only, whereas rural dwellers have both uses. Throughout the urbanization gradient, the different ethnic groups investigated here name and classify the existing intraspecific fruit variation using mainly morphological and organoleptic criteria. Interestingly, the most valued fruit criteria vary by use type: taste for self-consumption and purchase, size for sale. They also differ according to the groups of owners. People in rural sites, and especially in the Bassa group, tend to prefer larger fruits. This preference influences the seed selection process, with rural Bassa owners also favoring fruit size over fruit taste.

Divergences between rural and urban dwellers show how choices, preferences and strategies must be understood in a broad economic and social context, in which owners’ strategies are underpinned by different logics. Indeed, the choice of ethnovarieties responds to distinct motives along the urbanization gradient: City dwellers have few constraints, apart from lack of space, whereas rural dwellers are driven by demand and market requirements. Some owners are seeking a pool of different varieties, despite the call of market intermediaries for bigger fruits. Our study stresses the need to explore the consumption/production linkage to inform policy decisions on agricultural production and conservation strategies. It also suggests that urban areas should be considered not only for the threats they pose to local knowledge but also for the opportunities they offer as production areas able to foster knowledge exchanges. In the rural sites studied, located within the main production areas for African plum, the fact that some fruit phenotypes are favored could point to a possible negative impact on their diversity. However, the situation is offset by the species being highly outcrossing and by the limited removal of trees whose fruits are not valued. This calls for a methodology formally testing the intensity of varietal selection in commercial areas, and comparing it, along with preferences and propagation practices, in areas further away from commercial networks.

## Supplementary Information

**Additional file 1**. Description sheet for the morphological characterization of African plums.

## Data Availability

The data sets supporting the conclusions of this article are available in the OSF repository, https://osf.io/gp6ke/.
