# Supplementary material for: Shifting perceptions, preferences and practices in the African fruit trade: the case of African plum (Dacryodes edulis) in different cultural and urbanization contexts in Cameroon
Source: J Ethnobiol Ethnomed. 2021 Nov 8;17:65. doi: 10.1186/s13002-021-00488-3 (PMC8576868; doi:10.1186/s13002-021-00488-3)

Size

1) Very small <3 cm
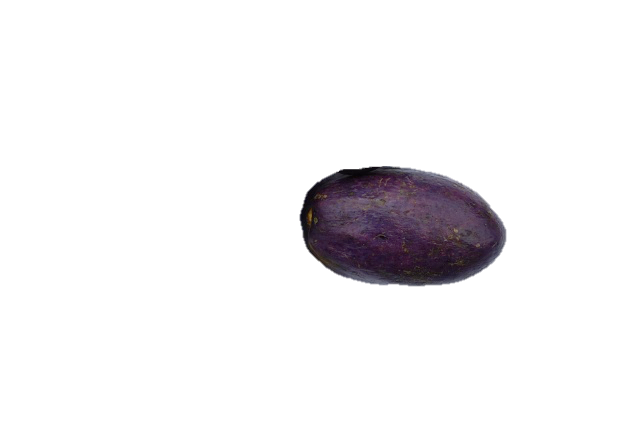


2) Small 3-5 cm
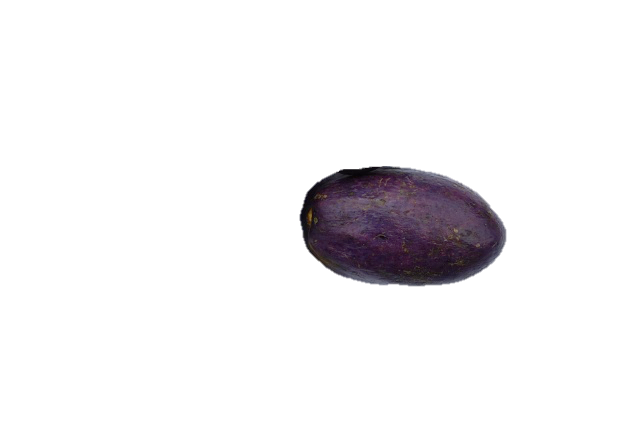


3) Medium-sized 5-7 cm
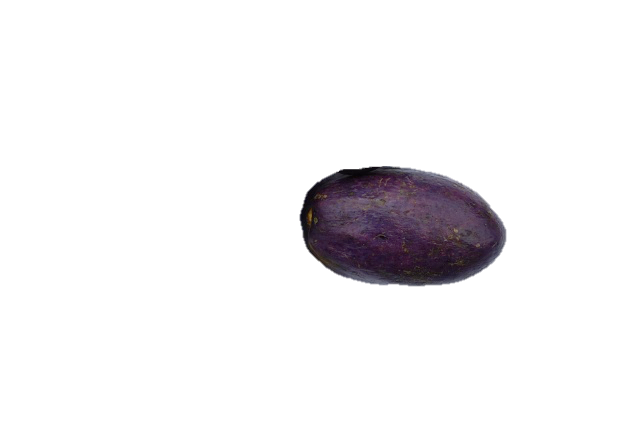


4) Big 7-10 cm
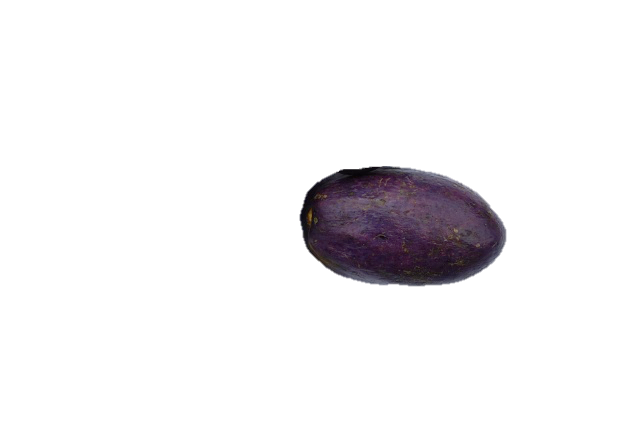


5) Very big >10cm
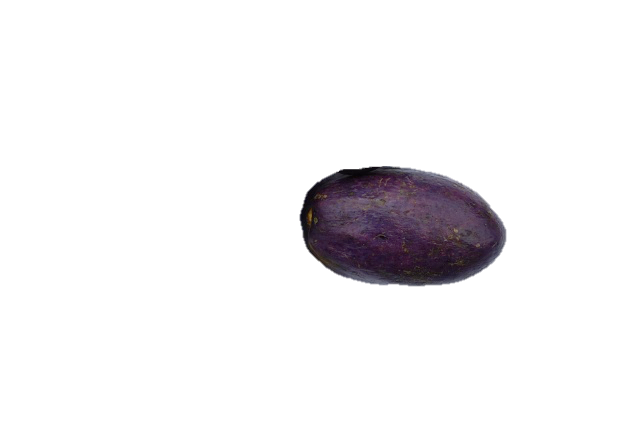


Skin color (ripe fruit)


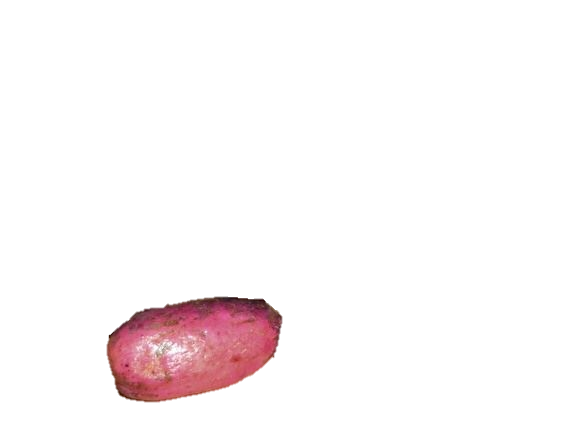

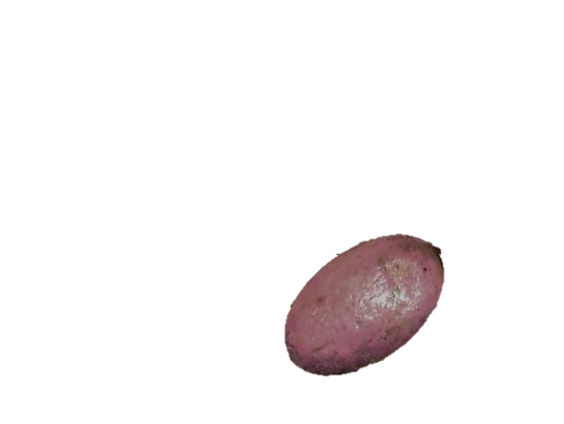
1) Pinky
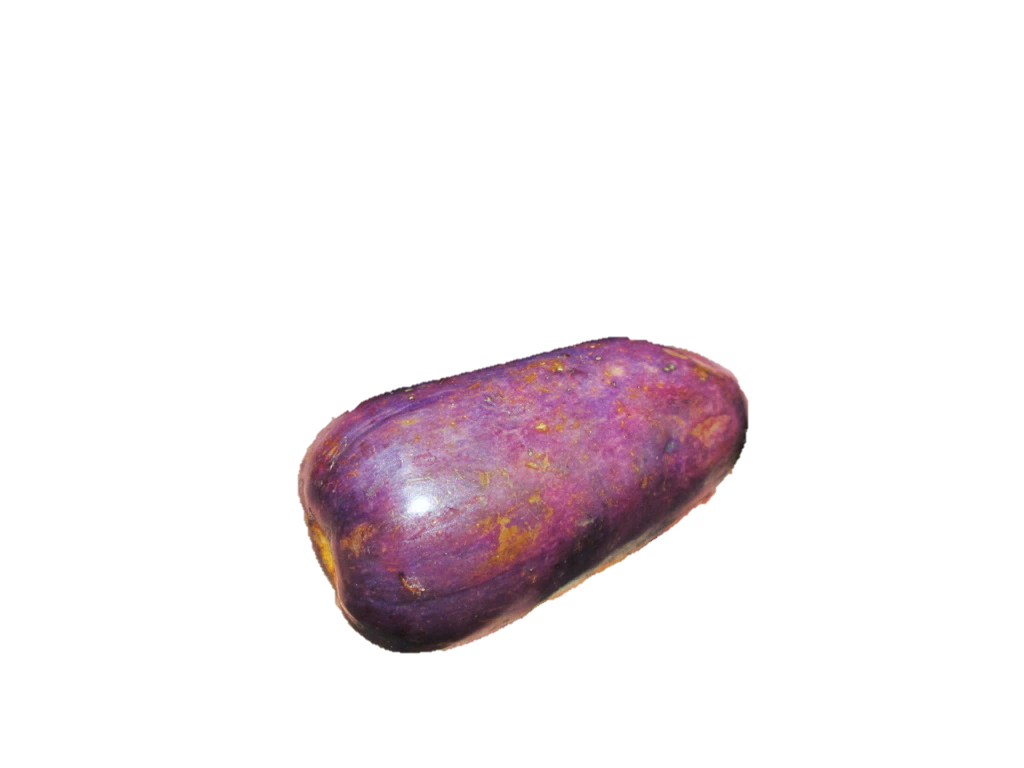


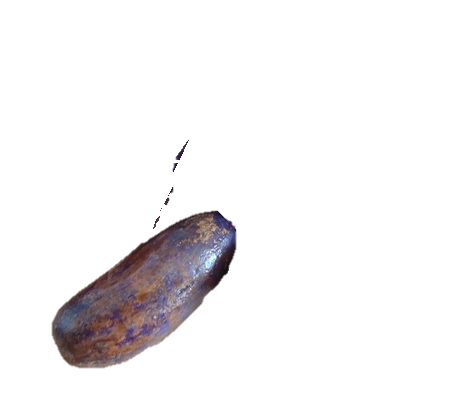

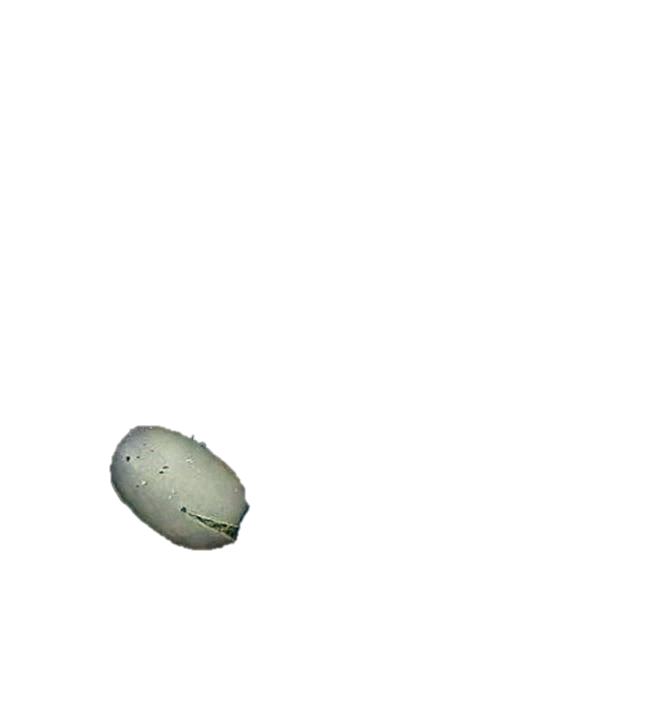


2)
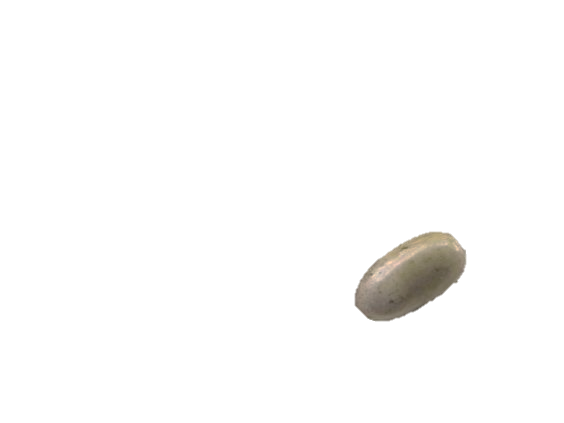
Whitish


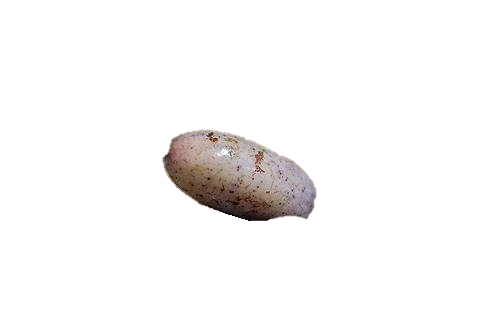


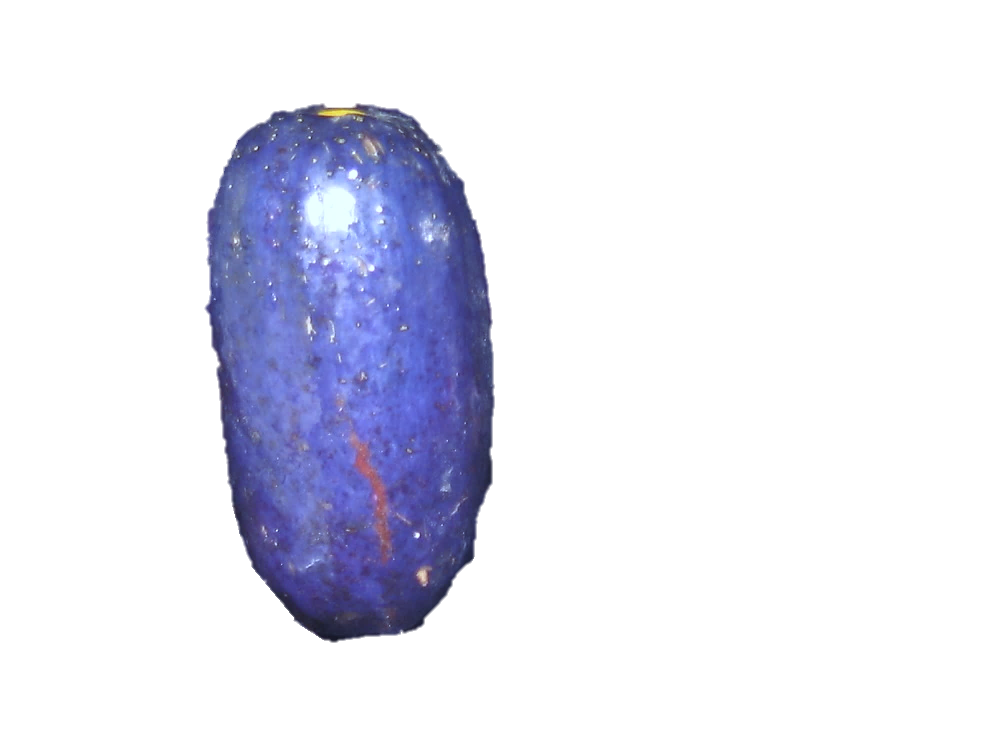

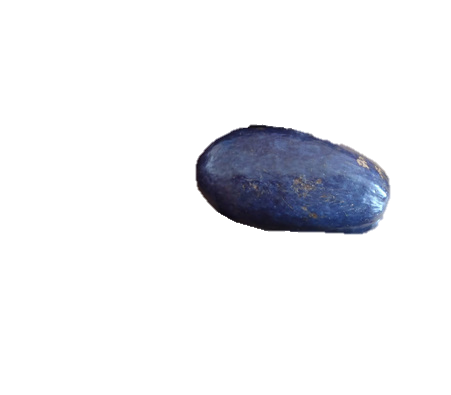
3) Blue, dark blue


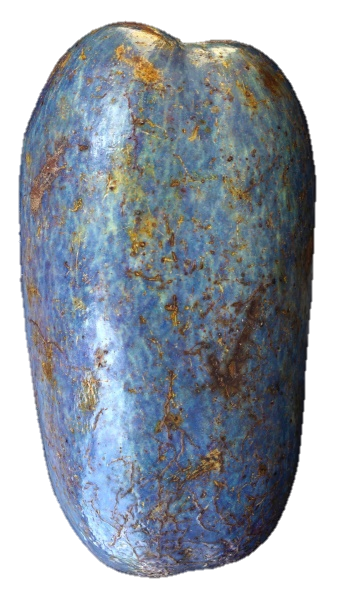

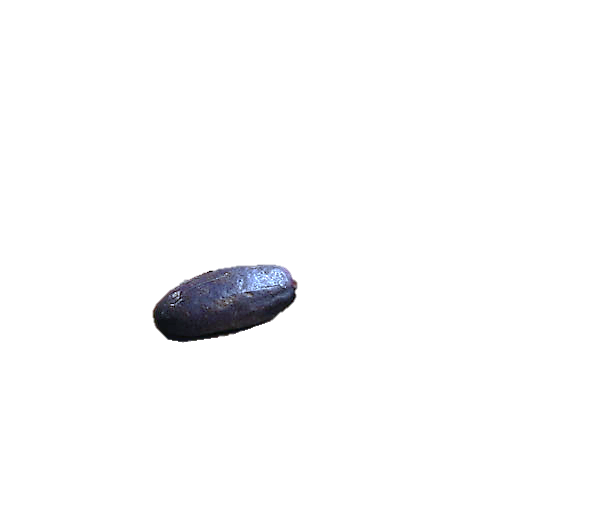


4)
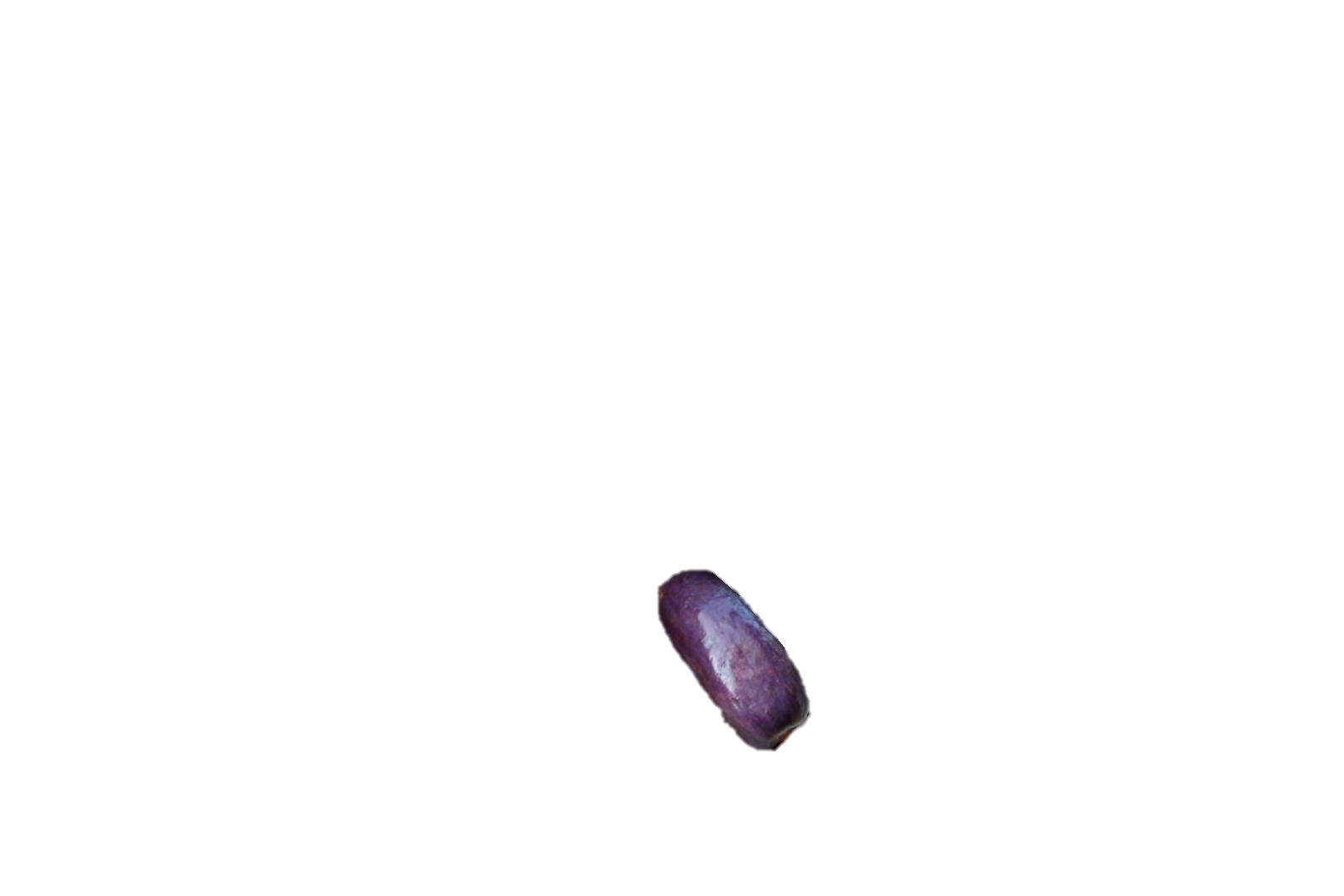
Purple


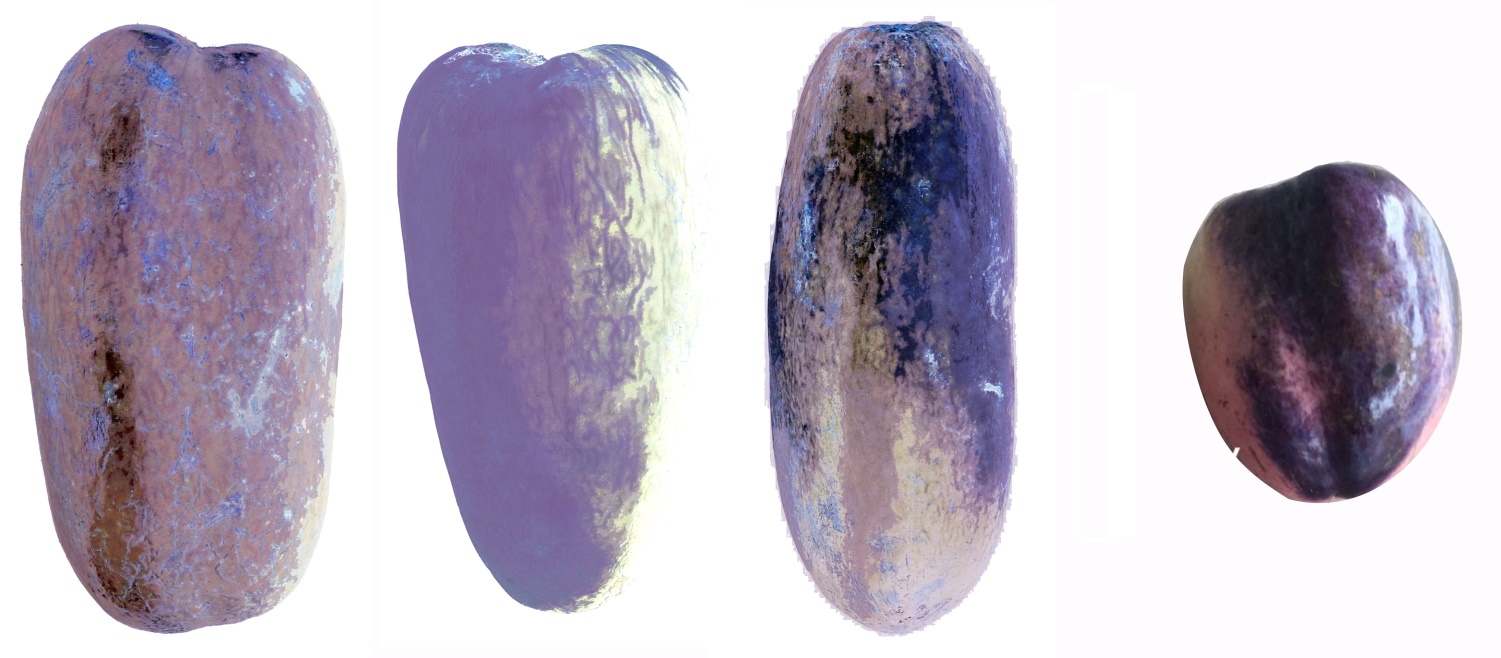


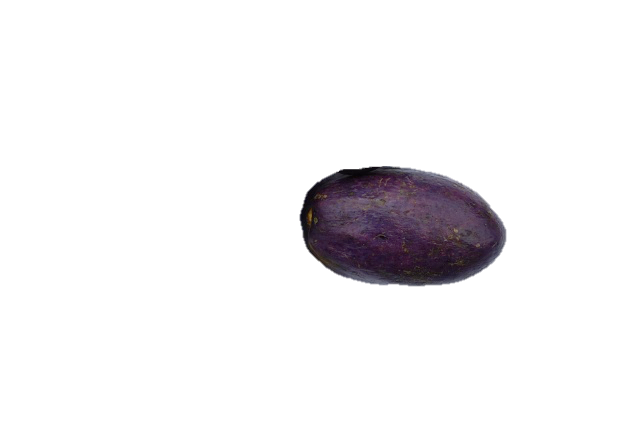


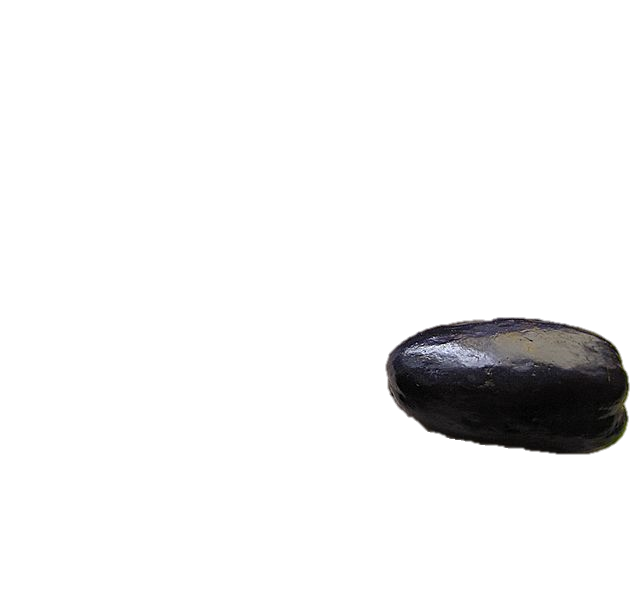
5) Black


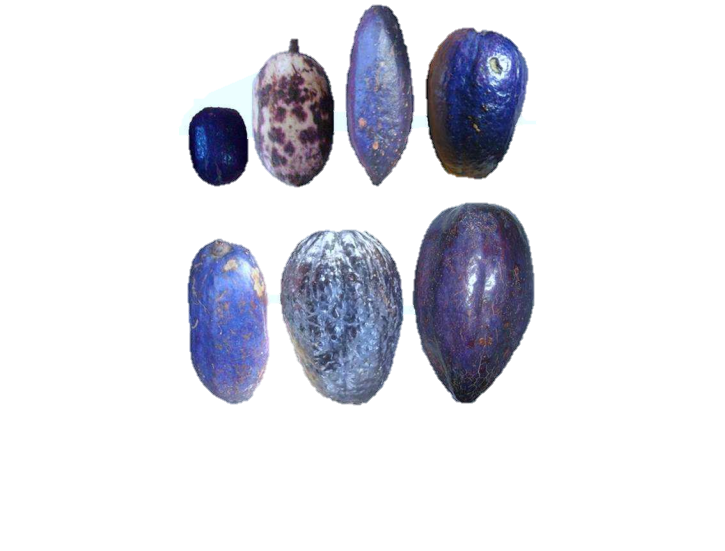
6) Two-coloured


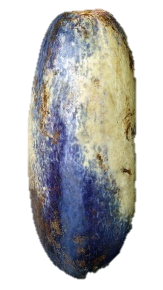


Pulp color


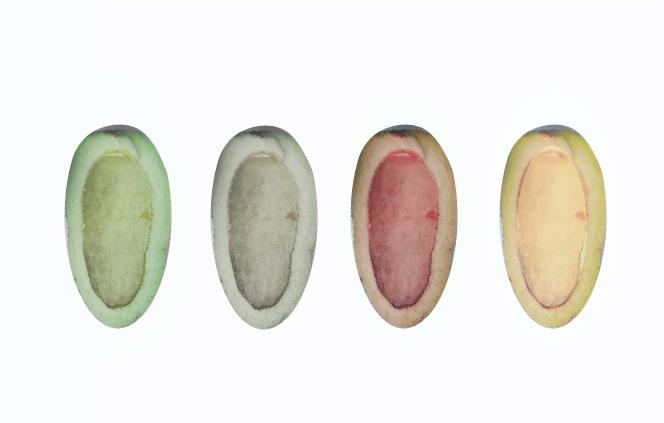


1) Green 2) White 3) Red 4) Yellow

Shape


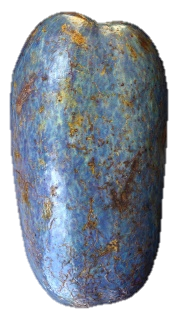

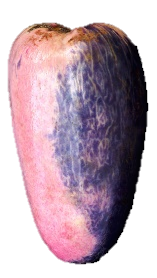

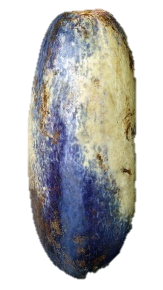

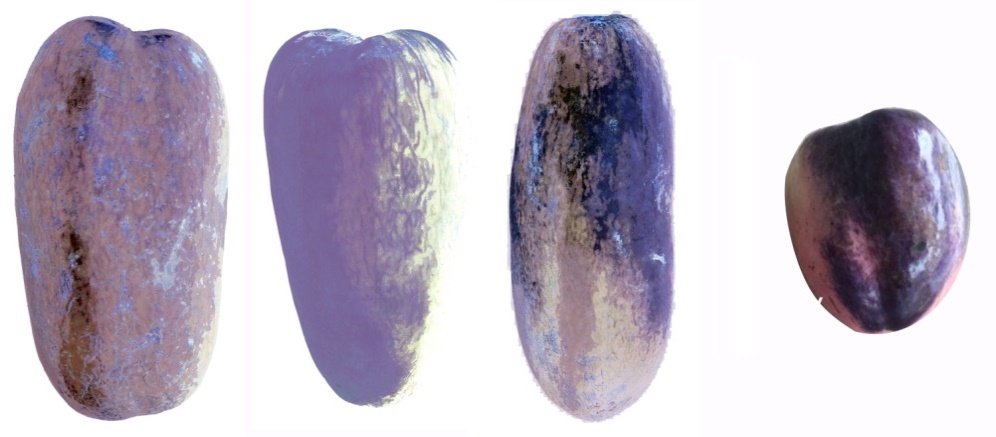


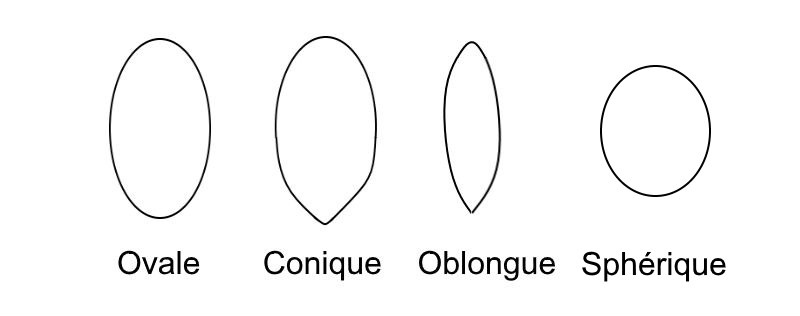

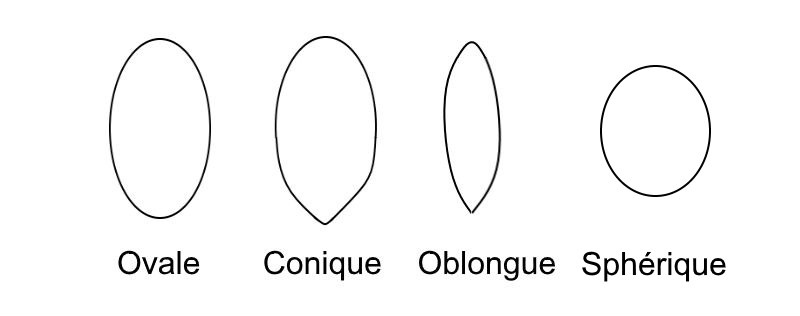

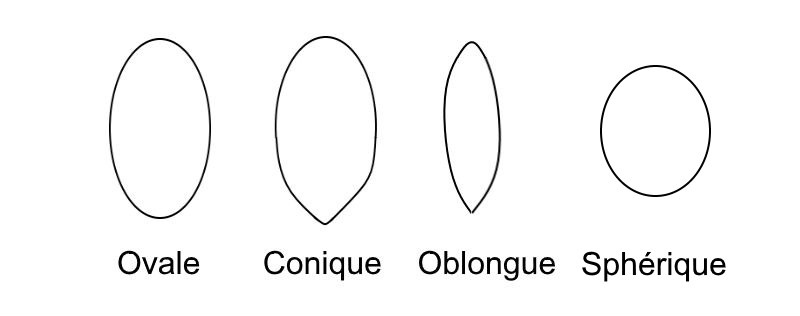

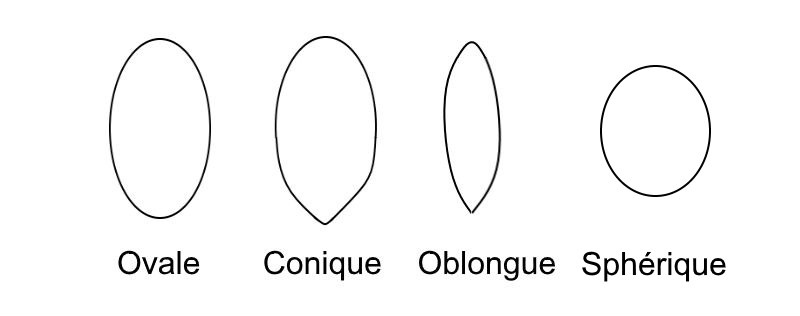


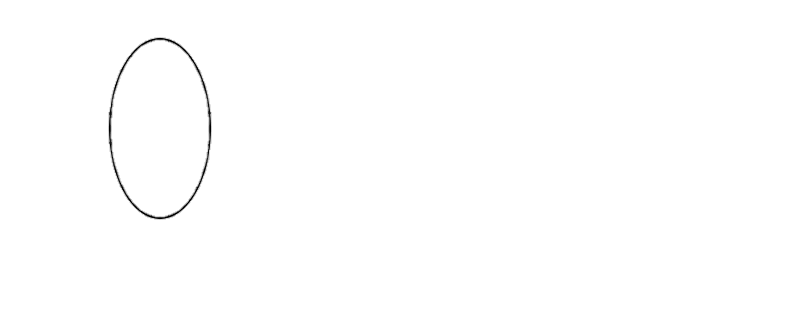

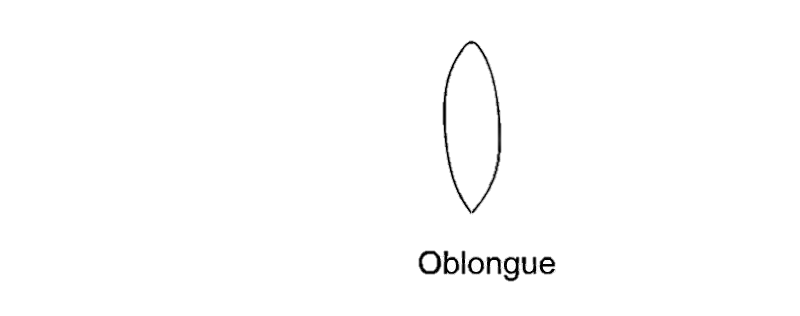


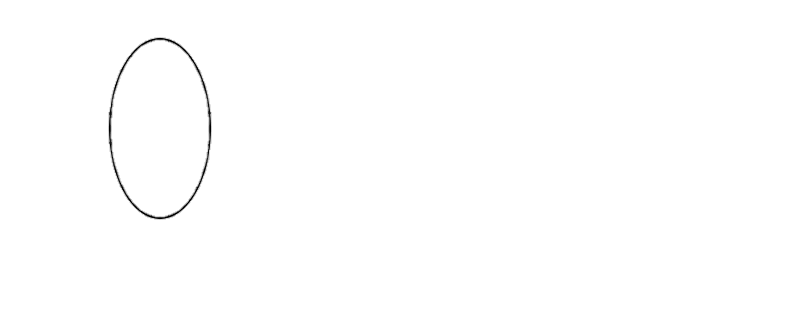

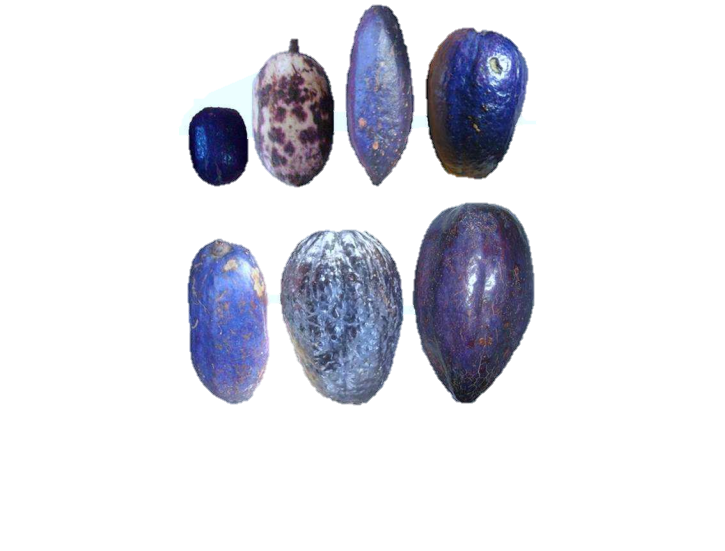


1) Oval 2) Conical 3) Oblong 4) Spherical


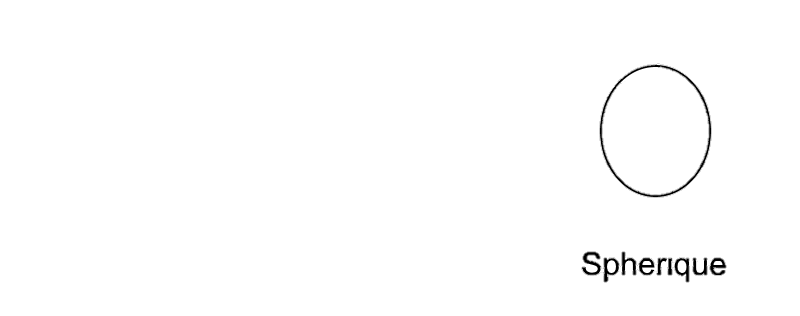


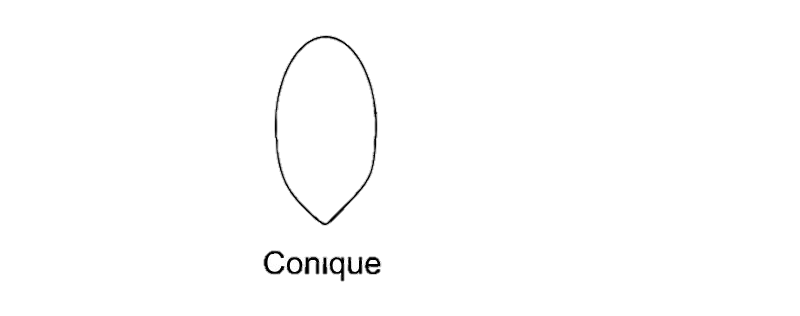


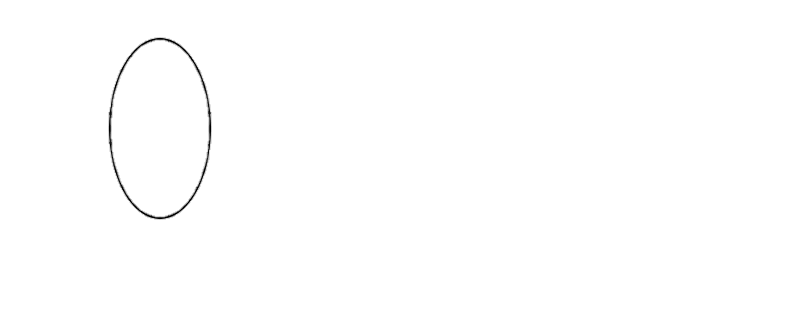


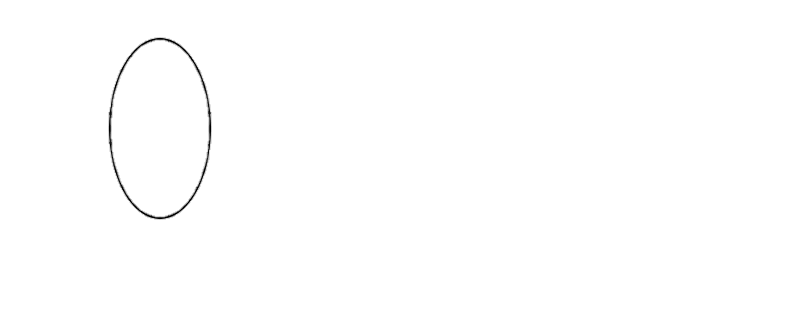

Supplement: Supplementary file 1 — Additional file 1. Description sheet for the morphological characterization of African plums. [file 13002_2021_488_MOESM1_ESM.docx]
